# Butterfly dichromatism primarily evolved via Darwin's, not Wallace's, model

**DOI:** 10.1002/evl3.199

**Published:** 2020-10-23

**Authors:** Wouter van der Bijl, Dirk Zeuss, Nicolas Chazot, Kalle Tunström, Niklas Wahlberg, Christer Wiklund, John L. Fitzpatrick, Christopher W. Wheat

**Affiliations:** ^1^ Department of Zoology Stockholm University Stockholm SE‐10691 Sweden; ^2^ Department of Zoology University of British Columbia Vancouver BC V6T 1Z4 Canada; ^3^ Department of Environmental Informatics Philipps‐University of Marburg Marburg DE‐35032 Germany; ^4^ Department of Biology University of Lund Lund SE‐22362 Sweden; ^5^ Department of Ecology Swedish University of Agricultural Sciences Uppsala SE‐75007 Sweden

**Keywords:** Butterfly, color, comparative analysis, dichromatism, phylogenetic ridge regression, phylogeny, sex, sexual dimorphism

## Abstract

Sexual dimorphism is typically thought to result from sexual selection for elaborated male traits, as proposed by Darwin. However, natural selection could reduce expression of elaborated traits in females, as proposed by Wallace. Darwin and Wallace debated the origins of dichromatism in birds and butterflies, and although evidence in birds is roughly equal, if not in favor of Wallace's model, butterflies lack a similar scale of study. Here, we present a large‐scale comparative phylogenetic analysis of the evolution of butterfly coloration, using all European non‐hesperiid butterfly species (*n* = 369). We modeled evolutionary changes in coloration for each species and sex along their phylogeny, thereby estimating the rate and direction of evolution in three‐dimensional color space using a novel implementation of phylogenetic ridge regression. We show that male coloration evolved faster than female coloration, especially in strongly dichromatic clades, with male contribution to changes in dichromatism roughly twice that of females. These patterns are consistent with a classic Darwinian model of dichromatism via sexual selection on male coloration, suggesting this model was the dominant driver of dichromatism in European butterflies.

**Impact Summary**

Males and females of many species are dimorphic; there are differences in the way the sexes look and function. One of the most studied types of dimorphism is dichromatism, where males and females have different colors. It is often assumed that sexual selection is important to dichromatism, as choosy females often mate with colorful males. At the same time, natural selection by predators against elaborated colors can especially be strong for females, as they may need to carry eggs or provide maternal care making them more vulnerable. For as long as we have known about natural and sexual selection, however, it has been debated which of these two forces initially creates dichromatism. Charles Darwin argued that sexual selection drives male color away from female color, whereas contemporary Alfred Russel Wallace instead thought that natural selection pulled female color away from the male's. Here, we revisit this debate using butterflies, one of the taxa Darwin and Wallace argued over, to determine whether Darwin's or Wallace's model is more important in the evolution of dichromatism. We used drawings from a field guide to quantify the color difference between males and females of all European non‐hesperiid butterfly species, and modeled how their colors have evolved over time. We show that the color of males generally evolves faster than that of females. By using the direction of male and female color evolution along the phylogeny, we also determined that changes in male color are around twice as important to dichromatism evolution than changes in female color. These results show that directional selection on males, likely due to sexual selection, is the main driver of dichromatism in butterflies. This supports Darwin's, but not Wallace's, model of dichromatism evolution, resolving a 150‐year‐old argument.

Sexual dimorphism, where the female and male on average differ in a trait, is commonplace in nature. The differentiation of reproductive roles has caused many different traits to diverge between the sexes, from basic metabolic functions and the immune system to behavior (International Mouse Phenotyping Consortium et al. 2017). Sex is a rich source of intraspecific variation, and sexual selection has produced some of the most striking phenotypes we know (Andersson 1994). Conspicuous colors in general, and sexual dichromatism (sexes differing in color) in particular, proved to be early problems for Darwin's theory of natural selection, as no obvious advantage seemed to be gained by having them (Kottler 1980). Darwin therefore expanded his other theory, sexual selection, to include the elaboration of traits through selection by female preference for conspicuous males (Darwin, 1871). The primacy of sexual selection in driving the exaggeration of male coloration has become a dominant view among evolutionary biologists (Badyaev and Hill 2003) and in textbooks (Zimmer and Emlen 2015). However, disentangling whether a contemporary observation of sexual dichromatism was caused by selection on either males or females is challenging, necessitating detailed study of the evolutionary history of how dichromatism evolved.

The evolution of dichromatism has arguably received the largest research effort in birds. Although a common assumption is that bird coloration has primarily evolved via the Darwinian model, the role of natural selection on female color evolution appears to have made at least as important a contribution (reviewed in Badyaev and Hill 2003). Evidence from studies on avian hormones suggests a colorful and monochromatic ancestry, from which dichromatism evolved through dulling of the female (Kimball and Ligon 1999). A recent study across all passerines, which comprises nearly 60% of all bird species, found that both natural and sexual selections were important to color evolution, but all identified drivers more strongly affected female than male color (Dale et al. 2015). Similar results pointing to natural, rather than sexual, selection in generating dichromatism have been found in fairy wrens (Johnson et al. 2013), blackbirds (Irwin 1994), and starlings (Maia et al. 2016), although not tanagers (Shultz and Burns 2017) or tyrant flycatchers (Cooney et al. 2019). In sum, in the taxon best studied, Darwin's model for dichromatism appears to be the exception rather than the rule, calling into question the general assumption that sexual selection is the primary agent generating dichromatism in other colorful clades.

If Darwin's model does not describe the majority of dichromatism evolution in birds, what model does? Wallace (1889) posited an alternative mechanism to Darwin's theory of sexual selection, arguing that female preference on small differences between males was unlikely to provide strong enough selection for dimorphism to arise. He noted that color clearly had strong implications in defense against predators through warning coloration, mimicry, and crypsis, and that the brooding of females typically put them under a higher risk of predation than males. Wallace therefore envisioned natural selection more likely to create dichromatism by favoring females to become drab and thereby cryptic.

Although Wallace's objections about the role of female preference have largely been disproven (Fisher 1930; Andersson 1994), his evolutionary path to dichromatism remains an important alternative hypothesis to sexual selection in generating dichromatism (Kottler 1980; Badyaev and Hill 2003; Kunte 2008). In the Darwinian model, natural selection is stabilizing on males, with directional sexual selection creating dichromatism via male coloration. In the Wallacean model, stabilizing sexual selection maintains the colorful males, whereas directional natural selection creates dull females. Importantly, the observation of brightly colored males and duller females alone cannot differentiate between the Darwinian and Wallacean scenario of the evolution of dichromatism, even though we often assume and teach the Darwinian model when dichromatism is observed; a reconstruction of the evolutionary history of color change is required (Kunte 2008).

Outside of birds, dichromatism is often assumed to be the result of the Darwinian model, this, however, is not often directly tested outside of a few notable exceptions. Nuptial coloration in African cichlid fishes is likely the result of strong sexual selection on males, as the hue of males of promiscuous species changes rapidly across speciation events (Seehausen et al. 1999). In Hawaiian damselflies, dichromatism has been suggested to be mainly caused by natural selection on both sexes (Cooper et al. 2016). A particularly interesting case is the polymorphic poison dart frog *Oophaga pumilio*, where both sexes have striking aposematic coloration, but directional sexual selection is the likely cause of the males being more brightly colored than females (Maan and Cummings 2009).

Butterfly dichromatism, while equally involved in the early debates between Wallace and Darwin (Smith, 1867), has received much less attention than dichromatism in birds. Although butterflies have become a model for understanding color evolution in general, and mimicry (Jiggins et al. 2001) and color development (McMillan et al. 2002) in particular, much less is known about their evolutionary history of dichromatism. Kunte (2008) investigated patterns of female‐limited Batesian mimicry in *Papilio*, one of the central butterfly clades in the original Wallace and Darwin dispute, and found them to be Wallacean. Oliver and Monteiro (2011) studied *Bicyclus* and *Junonia* and found both modes of evolution to be important. Although both these studies looked at well‐understood aspects of wing coloration, they were limited in scope to specific genera. More importantly, Kunte (2008) specifically investigated *Papilio* as he expected them to exemplify the Wallacean model. Thus, patterns of dichromatism across butterflies provide a currently untapped, rich opportunity for a large‐scale, unbiased study to assess the relative importance of Darwinian versus Wallacean color evolution.

Here, we present a large‐scale comparative analysis of color variation in butterflies, investigating the evolution of color across all European butterfly species by quantifying and comparing the evolution of dichromatism in males and females. Based upon the findings of color evolution in birds, we predicted that the Wallacean model has played a significant role in the evolution of butterfly dichromatism.

## Methods

### ANALYSIS OF COLOR

Butterflies have a much greater diversity in spectral sensitivity than birds, which varies dramatically across species and even between sexes (Frentiu and Briscoe 2008; Lebhardt and Desplan 2017). Thus, constructing a “butterfly” view of the world for a large‐scale comparative analysis is not possible. Here, we instead use a single perceptual perspective for analysis. We obtained depictions of all European non‐hesperiid butterflies by scanning the hand‐drawn illustrations from the butterfly field guide by Tolman and Lewington (1997), using the EPSON Perfection 4490 Photo Scanner with 1200 dpi and 24 bit in the RGB color spectrum (Zeuss et al. 2014). The obtained images were read into the R statistical programming environment (R Core Team 2019) for analysis, with general computational tasks aided by the additional R packages *tidyverse* (Wickham et al. 2019), *ape* (Paradis and Schliep 2019), *ggtree* (Yu et al. 2017), and *furrr* (Vaughan and Dancho 2018). We sampled 50,000 pixels from each image. For most species (*n* = 341), we sampled separate drawings for males and females, but for some monochromatic species (*n* = 52) only one drawing was included in the field guide and for these species we sampled the same image twice, once for each sex. We converted the obtained RGB values for each pixel to CIELAB (Lab) coordinates. The Lab coordinates code each color along a lightness axis (*L*) and two color axes, green to red (*a*) and blue to yellow (*b*). Unlike RGB, Lab has been designed to be approximately perceptually uniform for human vision. This means the three axes share a common perceptual unit, are linear, and allow for unbiased estimation of average colors. Note that the use of a human‐centered color space is intentional because the drawings were created for and by the human visual system. The Euclidean distance between two colors in Lab space describes how different the sensation of color (to the human eye) is for these points, independent of the location in color space. As the Euclidean distance in Lab space is the quantity of interest when comparing colors, our analyses will not rescale the color axes. This distance is often denoted ΔE, but we will write it as the vector magnitude $∥\vec{D}∥$ for reasons presented further below.

**Figure 1:**
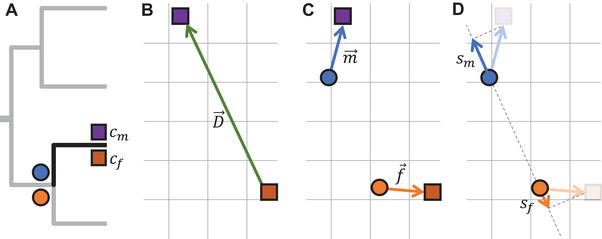
Graphical depiction of the analysis framework we used to study the evolution of dichromatism. (A) Imagine a dichromatic species with known colors shown by squares of male and female color, for which the ancestral phenotype has been estimated (shown in circles). (B) The vector between the male and female color describes the magnitude and orientation of their dichromatism ($\vec{D}$ is the dichromatism vector). Note that color is depicted in two dimensions for clarity, but all analyses are in three‐dimensional color space. (C) Vectors from ancestral to extant colors for each sex quantify the rate and direction of evolution ($\vec{m}$ and $\vec{f}$ are the sex‐specific rates of color evolution). (D) Projections of ancestral color for each sex onto the dichromatism vector, $\vec{D}$, allow for the quantification of sex‐specific contributions to D (s = sex specific contributions of dichromatism), disentangling male from female contributions to changes in dichromatism.

**Figure 2:**
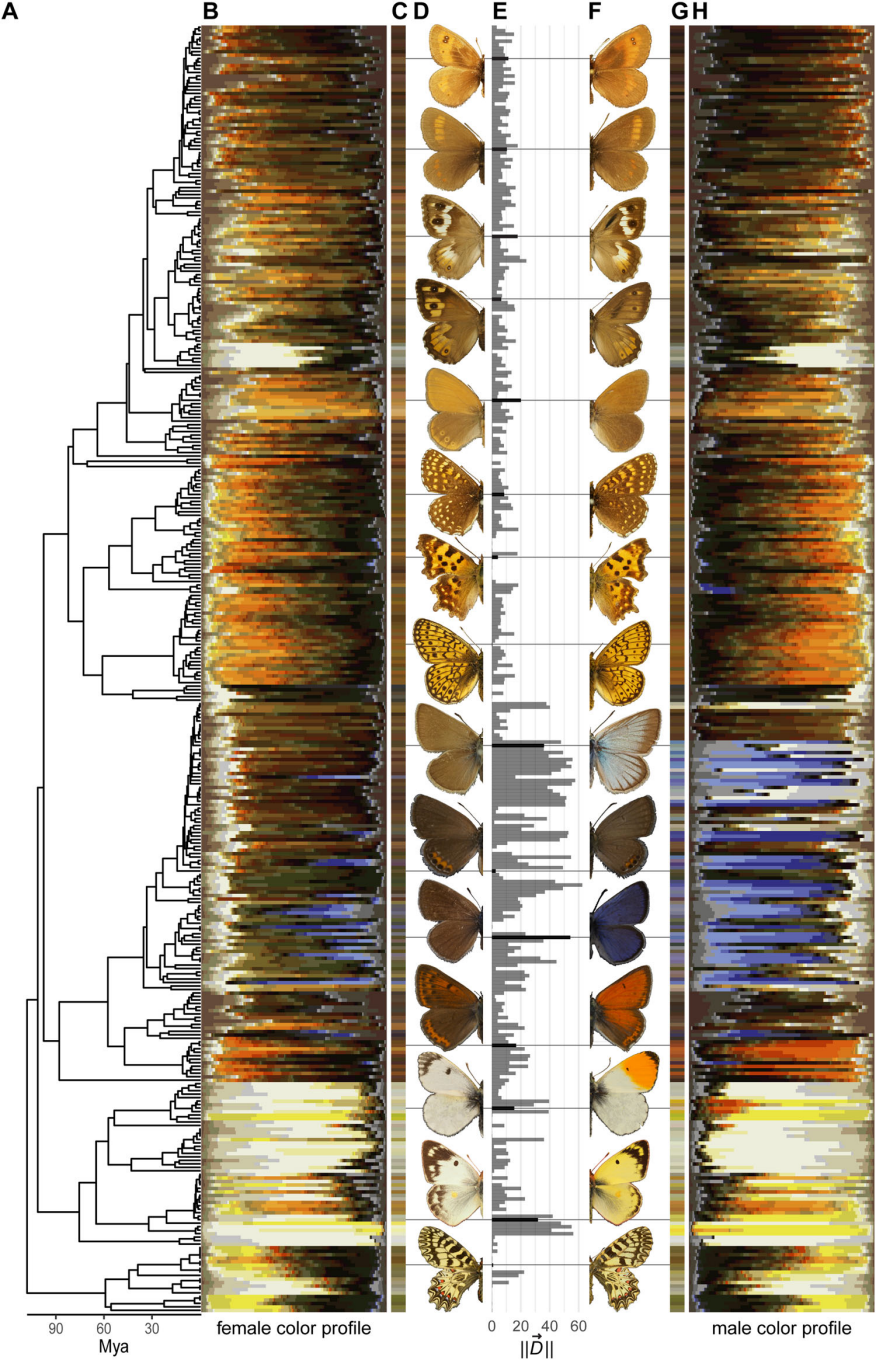
Dorsal wing color by sex of European butterflies. Panel A depicts the phylogenetic relationships between species. Panels B and H illustrate the color profiles of each sex, respectively, for each species as the fraction of pixels in each of 50 color clusters. Panels C and G give the female and male color centroids (average color). Panel E plots the distance between the female and male color centroids, used as the metric for dichromatism. Panels D and F show exemplar wings for males and females of the species that are connected by lines to the other panels. From top to bottom: *Erebia rhodopensis*, *Erebia pharte*, *Pseudochazara anthelia*, *Hipparchia semele*, *Coenonympha glycerion*, *Melitaea diamina*, *Polygonia c‐album*, *Boloria eunomia*, *Polyommatus damon*, *Freyeria trochylus*, *Glaucopsyche alexis*, *Lycaena hippothoe*, *Anthocharis cardamines*, *Colias hyale*, and *Zerynthia polyxena*. Photo credit: Kalle Tunström.

From the samples of color coordinates for each sex and species, we calculated centroids, or the average color of the sample (Figs. 2C and 2G) to summarize each image. Although the metric is simple, we found that it best captured the overall color difference between sexes across the phylogeny, as compared to alternative metrics of color overlap such as the volumetric intersection in color space, nearest neighbor distances, or differences in discrete palette use (see Supporting Information). Additionally, we calculated the Euclidean distance between the centroids of the two sexes within each species (Figs. 1A, 1B, and 2E), to be taken as the magnitude of dichromatism within that species. Importantly, and to highlight the power of our approach compared to previously used metrics of color overlap, the use of centroid colors allowed for direct modelling of the male and female phenotype on the original axes, from which the evolutionary history of dichromatism can then be inferred.

We focused our analyses on the dorsal sides of the butterflies (i.e., the top side), as these have greater variability and a wider range in dichromatism, yielding more statistical power. All analyses were repeated for the ventral sides, which are reported in the Supporting Information. For visualization purposes (Figs. 2B and 2H), we classified all sampled pixels into a shared palette of 50 colors using *k*‐means clustering. We ordered the clusters by treating it as a Travelling Salesman Problem solved by arbitrary insertion followed by two‐edge exchange improvement.

The phylogenetic signal of dichromatism was estimated by fitting an intercept only phylogenetic linear model with the R package *phylolm* (Ho and Ane 2014), and estimating Pagel's *λ*. A confidence interval was obtained through bootstrapping (*R* = 1000).

### EVALUATION OF COLOR ACCURACY

To evaluate whether the drawings from the field guide yielded a good representation of the true color differences between species and sexes, we additionally took photographs of a smaller subset of species for comparison. To obtain a good cover across both the phylogeny and different levels of dichromatism, we employed a stratified sampling strategy. We divided the species list by family and split the species within each family in half based on the dichromatism score described above. We then randomly sampled up to 10 species from each category. Using the resulting list of species, color‐standardized photographs were taken of a specimen of each sex, depending on availability in the collection of the Swedish Museum of Natural History. Specimens were selected based on condition, with preference for males and females from the same locale. The specimens were kept in wooden drawers, with a tightly sealed glass lid, kept in cabinets protected from light. However, because they were donated by collectors, storage prior to being donated to the museum is unknown. All specimens appeared to be in good condition with intact wing scales, although some bleaching was noticeable for some of the specimens. This procedure resulted in photographs for 53 species.

Photos were taken with an Olympus (Tokyo, Japan) E‐M1MarkII with an Olympus (Tokyo, Japan) M.Zuiko Digital 60‐mm f2.8 macro lens, used on a stand at a distance of approximately 16 cm from the specimen. The specimen was lit with two spotlights and a dual‐armed LED light microscopy light source, diffused through white paper. All photos were taken at an ISO of 320 and 60‐mm focal length, with the aperture and shutter speed being adjusted on a per session bases using a CameraTrax (Menlo Park, CA) 24 color card, to adjust for slight variation in environmental light. This color card was also used to standardize the colors between photographs in Adobe Lightroom.

All backgrounds were removed from the photographs using Adobe Photoshop, after which the images were read into R. Using the same procedure as for the drawings, we calculated color centroids for each image. We note that it is not necessary for the drawings to accurately reflect the color directly, but rather they should accurately represent the relative color differences (i.e., distances) between specimens. To evaluate whether the structure in color space between sexes and species as derived from the drawings is similar to the structure derived from photographs (Bergeron and Fuller 2018), we calculate the pairwise differences among each of the 106 samples. These differences were found to correlate well between the two datasets, with Pearson correlation coefficient (*r*) of 0.87, 0.85, and 0.78 for the *L*, *a*, and b axes, respectively (Fig. S1). Color differences were larger in the drawings than in the photos (regression slopes were 0.54, 0.44, and 0.57), which could be caused by, for example, bleaching of specimens or an exaggeration of color differences in the drawings, but this absolute difference does not affect our analyses.

### PHYLOGENY

We used the complete phylogeny of European butterflies from Wiemers et al. (2020). A full description of the data and methods used in the creation of this phylogeny can be found there. Briefly, the tree was generated by grafting European clades onto a time‐calibrated backbone, which included about 50% of extant butterfly genera (Chazot et al. 2019). This avoided potentially strong biases when estimating topology and divergence times from very asymmetrically sampled taxa. The backbone was taken from a recent reevaluation of the timing of divergence of butterflies (Chazot et al. 2019) based on fossil and host‐plant age evidence. The European butterflies that needed to be added to the tree were divided into 12 subclades. For each subclade, a tree reconstruction without time calibration (only estimating relative branch lengths) was performed. The subclade trees were then rescaled using the ages estimated in the backbone and were subsequently grafted on to the backbone. For this study, we performed our analyses on the Maximum Clade Credibility tree obtained from the posterior distribution of grafted trees (Wiemers et al. 2020).

### MODELLING OF COLOR HISTORY AND EVOLUTION

To infer the evolutionary history of the male and female centroid along the phylogeny, as well as the distance between them, we employed a recently developed ridge regression method (Castiglione et al. 2018), as implemented in the R package *RRphylo*. This method employs a series of penalized regressions to model the evolution of traits through multivariate space along the branches of the phylogeny. Using this algorithm, we modeled male and female centroid color evolution independently across European butterflies. In contrast to other methods, such as Brownian motion models, rate variation along all branches can be estimated. Normally, this large number of rates leads to overparametrization (Kratsch and McHardy 2014); however, *RRphylo* additionally minimizes the variance in evolutionary rates. At each branch of the phylogeny, the ridge regression estimated an ancestral trait value, that is, a male and female color centroid in three‐dimensional color space (Figs. 1A and 1C). Additionally, regression slopes at each branch represent two vectors in three‐dimensional color space describing the rate and direction of color evolution (Fig. 1C). We note that we do not expect a mean‐variance relationship in the color trait, where the rate of color evolution would be higher for “larger colors,” and we therefore do not perform standardization of the trait before analysis. A visual explanation of the quantities modeled can be found in Figure 1.

We chose this method so we could model our phenotypes in the original continuous color space instead of using categorizations. Thus, we can avoid using any arbitrary dichotomization of colors into categories such as All elaborated and cryptic, attempts at which resulted in unacceptable levels of subjectivity. Our current functional understanding of butterfly coloration is insufficient to categorize colors as elaborated, and the lack of data on typical background color prevents objective distinction between cryptic and conspicuous types. Critically, the approach we used allowed us to simultaneously estimate male and female phenotypes, male and female rate, and direction of evolution, as well as the magnitude and direction of dichromatism. This enabled us to clearly differentiate the roles of males and females in the evolution of dichromatism, free of assumptions about color function. Note that our method presented here has broad applicability also for other studies of dimorphism.

#### Ancestral state estimation of dichromatism

Using the *RRphylo*‐estimated ancestral trait values across the tree for both males and females, the ancestral dichromatism at each node was derived. Let the male color centroid be $c_{m}$ and the female color centroid be $c_{f}$. Draw a vector $\vec{D}$ from $c_{m}$ to $c_{f}$. Then the magnitude (or length) of this vector, denoted $∥\vec{D}∥$, is equal to the dichromatism metric ΔE, that is, the distance between centroids (Figs. 1A and 1B).

#### Rates of color evolution

In addition to the centroids $c_{m}$ and $c_{f}$, we obtained evolutionary vectors $\vec{m}$ and $\vec{f}$. These vectors describe the local rate and direction of color evolution of males and females, respectively. The magnitude of these vectors describes the male and female rates of evolution and are measured as the change in units of Lab color space per million years. These rates have effectively arbitrary units because the Lab color space has arbitrary units. The ratio of mean magnitudes $(∥\vec{m}∥)/(∥\vec{f}∥)$ was used to compare male and female rates of evolution. Additionally, we related the sex‐specific evolutionary rates $∥\vec{m}∥$ and $∥\vec{f}∥$ to ancestral estimates of dichromatism $∥\vec{D}∥$ to test whether male or female evolutionary rates increased along dichromatic branches. We fitted a model of the form *log(male rate/female rate) ∼ dichromatism*. The ratio was log transformed so it becomes linear and symmetrical with regard to sex. Note that the slope in this model is exactly equal to the interaction term in *log*(*rate) ∼ dichromatism × sex*, because $\ln a-\;\ln b=\ln\frac{a}{b}\;$.

#### Male‐ and female‐driven changes in dichromatism

To gain additional insight into which sex was important for changes in dichromatism, we calculated the effective rate of change in dichromatism due to males and females and correlated this sex‐specific rate of change with the effective rate of change in dichromatism in the species. We defined the effective rate of change in dichromatism as the scalar projection of an evolutionary vector on $\vec{D}$, that is, the rate of the color change aligned with the existing direction of dichromatism. When this is positive, dichromatism is increasing and when it is negative species become more similar in color. We obtain the scalar projections for males and females, $s_{m}=\frac{\vec{m}\cdot \vec{D}}{∥\vec{D}∥}\;$ and $s_{f}=\frac{\vec{f}\cdot \vec{D}}{-∥\vec{D}∥}\;$, where · denotes the dot product. The species‐level effective rate of change in dichromatism is given by $s\;=\frac{\left(\vec{m}-\vec{f}\right)\cdot \vec{D}}{∥\vec{D}∥}\;$. Note that it is equivalent to the difference in $∥\vec{D}∥$ of the ancestral and derived phenotypes. As the changes in male and female color ($s_{m}$ and $s_{f}$) together create the change in dichromatism s, they are expected to each contribute exactly half of the magnitude of s only if male and female contributions are equal. A linear regression of $s_{m}∼\;s$ is in this case expected to yield a slope of 0.5. If male color evolution contributes more to changes in dichromatism than expected, $s_{m}$ should be more than 0.5 times the magnitude of s, and the regression slope should increase accordingly.

#### Significance testing

To evaluate whether our observed differences were statistically significant, we compared the relevant statistics with reference distributions obtained by permutations of our dataset. Specifically, we randomly swapped the sex labels for each species with equal probability (Bergeron and Fuller 2018). This permutation procedure maintains the observed phylogenetic structure and maintains the observed presence of colors in each species and clade, but permutes any sex‐specific signal. We applied the ridge regression method to 1000 permuted datasets, extracted the same statistics, and computed two‐tailed *P*‐values.

## Results

The color of European butterflies showed strong phylogenetic signal (Fig. 2), as does dichromatism (Pagel's *λ*, estimate [95% CI] = 0.79 [0.66‐0.86]). Typically, color evolution is shared between the sexes, as both the rate and orientation of evolution are closely aligned (Fig. S2). By comparing the lengths of the male and female evolutionary vectors, we investigated which sex has a higher rate of color evolution. As the length of the vector is the evolutionary rate, we calculated the ratio of magnitudes $∥\vec{m}∥/∥\vec{f}∥$. On average, male color evolved at a 26% faster rate than female color (Figs. S2 and S3; *P* < 0.001), suggesting that males are more likely to have contributed to sex differences in color.

**Figure 3:**
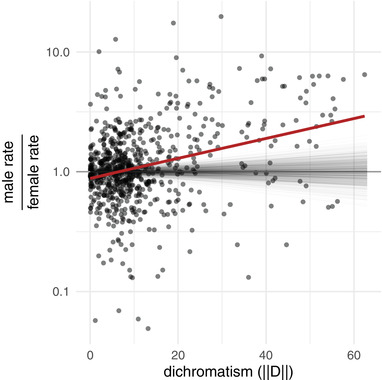
As dichromatism increases, the rate of color evolution becomes more male biased. Points show the ratio of male to female evolutionary rate in color evolution along branches of the phylogeny, in relation to dichromatism. The red line shows the observed relationship, whereas each thin gray line represents samples from the null‐distribution of that relationship. The null distribution was obtained by randomly permuting males and females within species along 1000 phylogenies. Note that the *y*‐axis is on a logarithmic scale and that 1 indicates equal rates.

Higher male rates of color change could be explained by a high volatility in male color that is independent of dichromatism. To assess this, we compared the ratio of rates to the level of dichromatism along the same branches. Evolutionary rates of color change become significantly more biased toward males as the estimated dichromatism along the same branch increases. This results in a strongly male‐biased (3:1) difference in evolutionary rate at highly dichromatic parts of the phylogeny, whereas male and female rates are roughly equal along monochromatic branches (Fig. 3; *P* < 0.001). This refutes the possibility of rapid male color evolution occurring orthogonal to dichromatism.

**Figure 4:**
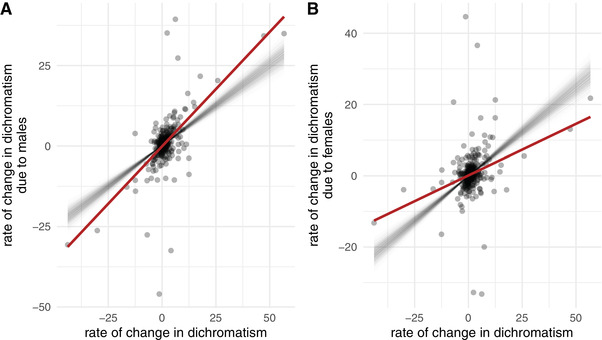
Changes in dichromatism are more likely to be the result of male change than female change. The *x*‐axis represents effective rates of change in dichromatism, for each species. The *y*‐axis shows the male (A) or female (B) attributable parts of that change along the same branch, which is the evolutionary rate of color evolution in the direction of dichromatism (see main text). The red line shows the observed relationship, whereas each thin gray line represents sample from the null distribution of that relationship. The null distribution was obtained by randomly permuting males and females within species along 1000 phylogenies. Note that the results in the two panels are not independent, the slopes necessarily add up to 1.

However, increased rates of male color evolution could represent color changes within highly dichromatic clades, rather than color changes that have caused increased dichromatism. Therefore, complementary to the rates of evolution, we quantified the direction of color change. Instead of expressing male and female color evolution relative to the color space itself, we redefine it in terms of their direction toward the other sex. These “effective” evolutionary rates can be calculated by projecting the evolutionary vectors on the vector of dichromatism (Fig. 1D). That is, instead of looking at the absolute size of color change, these projections only measure the magnitude of change along the direction of dichromatism and can therefore differentiate whether there is evolution occurring toward, perpendicular to, or away from the other sex. We also define a net change in dichromatism itself, by projecting the difference between male and female evolutionary vectors on the direction of dichromatism, and compare the contributions of males and females to changes in dichromatism. Evolutionary changes in dichromatism are over two times more strongly driven by male than by female color changes (Fig. 4; male slope = 0.71 and female slope = 0.29; *P* < 0.001). Similar results were obtained when excluding the highly dichromatic family Lycaenidae (results not shown). Thus, when dichromatism increases, it is typically the male that is evolving away from the female. When comparing closely related species that differ in dichromatism, the females are therefore expected to look roughly twice as similar as the males.

On the ventral sides, the color variation across the phylogeny is smaller than on the dorsal side, and dichromatism is typically much less pronounced (Figs. S4 and S5). Color in males still evolves faster than females, but the difference is smaller and not significant (male rate/female rate = 1.08, *P* = 0.082; Fig. S6). The difference between male and female rates is also not related to dichromatism (Fig. S6; *P* = 0.678). Nonetheless, also on the ventral sides, males disproportionally contribute to overall changes in dichromatism (Fig. S8; *P* = 0.002). Because the rates of evolution are similar between the sexes, this indicates that the direction of male evolution is more often aligned with the direction of dichromatism than the female direction on the ventral sides.

## Discussion

Male color evolution is on average faster than female color evolution in European butterflies. This difference in evolutionary rates is particularly strong in those parts of the phylogeny where dichromatism is high. Furthermore, along the branches where dichromatism is changing most markedly, males evolve faster along the direction of dichromatism than females implicating the directional evolution of males. Contrary to patterns found in birds, these results give no indication that it is common in butterflies for dichromatism to evolve due to female‐limited chromatic evolution under natural selection, as argued by Wallace. Rather, all analyses provide strong support for the Darwinian model of dichromatism, where dichromatic lineages generally result from strong directional selection on male elaboration, with the female retaining a more ancestral form, suggesting that sexual selection can significantly accelerate color evolution.

The evolution of dichromatism due to sexual selection could arise due to male‐male competition or female choice. Although male‐male competition could generate more elaborated coloration, there is no empirical evidence for this in butterflies, instead indicating physiological performance being of more importance (Kemp 2002; Kemp et al. 2006; Martínez‐Lendech et al. 2007). A limited number of studies investigating female choice have found them to prefer males with brighter coloration, where it was likely used as an indicator of age (Papke et al. 2007), or saturated coloration (Davis et al. 2007) used as an honest signal of quality (Davis et al. 2012). For butterflies, there are no large datasets available on measures of sexual selection or mate choice with broad phylogenetic coverage, making it difficult to integrate our results with the rich literature on butterfly ecology (e.g., Jiggins 2008; Kemp and Rutowski 2011). Future work should further consolidate proxy measures for sexual selection related to the patterns described here, or more directly measure sexual selection through, for example, Bateman gradients (Fritzsche and Arnqvist 2013).

It is clear that the dorsal sides of the butterfly wings are much more likely to be dichromatic than the ventral sides. Conflict between the different signaling functions of coloration, such as mate selection and antipredator functions, can be reduced by separating these patterns to different parts of the body (Endler 1992). It has long been suggested that dorsal and ventral wing patterns provide butterflies with the opportunity to separate functions and signals, as butterflies fold their wings at rest, hiding the dorsal wing surface from predators (Darwin, 1871; Wallace, 1889). Characters such as eyespots evolve faster and are more likely to exhibit sex‐biased rates of evolution on the dorsal surface than the ventral surface (Oliver et al. 2009). Our results align with the theory that sexual selection acts stronger on the dorsal side of the wing, although we still find support for Darwin's model in the less frequent instances that ventral surfaces become dimorphic.

By conducting this large‐scale comparative analysis of color evolution, we have generated an unprecedented dataset for formulating questions and setting the stage for future analyses. Color variation is highly clustering across butterfly species, capturing the distinctive patterns for which each of the families is well known (e.g., Pieridae are the whites and yellows and Lycaenidae are the blues). Interestingly, most families generate their dominant colors using different mechanisms, with Nymphalidae and Papilionidae relying primarily on ommochromes, Pieridae on pterins, and Lycaenidae on structural innovations for their metallic blue hues. Although frequent changes in color have occurred between species, within families these changes are generally limited to the use of a particular set of colors, suggesting significant constraints on the invasion of novel areas in the color morphospace. The rare appearances of novel colors along a branch of the phylogeny provide a rich set of outstanding questions, including whether the constraints are chiefly biochemical or ecological.

The evolution of sexual dimorphism is expected to be under significant evolutionary constraint due to intralocus sexual conflict, as males and females share the vast majority of the genome and are therefore expected to share the majority of loci controlling color. For sexes to diverge, the trait would first need to undergo genetic decoupling (Lande 1980; Poissant et al. 2010; Hermansen et al. 2018). Nonetheless, even in the face of apparent strong constraint, sexual dimorphism can evolve rapidly (Stewart and Rice 2018) and differ strongly between closely related species (Owens and Hartley 1998). In at least some cases, dichromatism may evolve by a simple molecular mechanism at a single locus (Gazda et al. 2020). Although it is clear that the majority of the color evolution in European butterflies is shared between the sexes, it remains an open question whether this reflects substantial unresolved antagonistic selection.

Here, we have analyzed color variation from a hand‐drawn field guide, similar to studies in birds (Dale et al. 2015). Importantly, we have also shown that color estimates from these drawings are well aligned with colors collected from photographs of museum specimens (Fig. S1). Nonetheless, field guide colors could differ substantially from true reflectance spectra, but both sources of data show a remarkably similar data structure in bird studies (Bergeron and Fuller 2018), and thus provide an objective first step in analysis that reveals strong evolutionary patterns. Our approach is also blind to ultraviolet coloration. However, given the datasets generated to date (Ghiradella et al. 1972; Rutowski et al. 2005; Kemp 2007), such patterns are likely to generate an even stronger male‐biased signal in support of Darwin's model. Future work that extends these analyses to include species from the other continents, ideally with standardized reflectance spectra, will be an important advance. Understanding the origins of the pervasive dichromatism observed across the diversity of animals is central to disentangling the relative contributions of sexual versus natural selection in generating much of the color variation we see in the natural world. The contrast between Wallacean dichromatism in many birds and Darwinian dichromatism in European butterflies invites the continued study of this question. This is a challenging endeavor, as we need to reconstruct the evolutionary history of diverse interactions that have generated the extant variation observed today. Butterflies represent a currently untapped resource for such investigation, given their species diversity as well as diverse mechanisms for generating color, resource and habitat use, signal‐receiver dynamics, and life history strategies. As a step toward exploiting this rich resource, here we not only present our findings on the evolution of dichromatism in European butterflies, but provide all steps of our analysis and results as a resource for such future integrative studies.

## AUTHOR CONTRIBUTIONS

The study was conceived by CWW. Data were collected by DZ, KT, and CWW. The phylogeny was contributed by NC and NW. The analyses were designed by WvdB, with help from DZ, JLF, and CWW. The analyses were performed by WvdB with help from DZ. All visualizations were by WvdB. The manuscript was written by WvdB and CWW, with input from all authors.

## DATA ARCHIVING

All data used in this work (color centroids, dichromatism values, and the phylogeny) as well as code to reproduce the analyses and figures can be found on GitHub (https://github.com/Ax3man/vdBijl_etal_EvolLett_2020) and is archived at Zenodo (https://doi.org/10.5281/zenodo.4037399).

**Associate Editor: L. Bromham**

## Supporting information


**Tables S1**. Centroids of males and females in Lab color space, and dichromatism (defined as the centroid distance)
**Figure S1**. Comparison between the structure in color space using either photographs or drawings for a subset of 53 species.
**Figure S2**. Male and female rates of butterfly color evolution.
**Figure S3**. Comparison of the observed ratio between male and female evolutionary rate.
**Figure S4**. Coloration by sex of European butterflies, on the ventral sides.
Figure S5. Male and female color evolution are strongly correlated, also on the ventral sides.
**Figure S6**. Comparison of the observed ratio between male and female evolutionary rate with the expected distribution from permuted phenotypes.
**Figure S7**. As ventral side dichromatism increases color evolution becomes evenly balanced between males and females.
**Figure S8**. Changes in ventral side dichromatism are more likely to be the result of male change than female change.
**Figure S9**. The phylogenetic tree as used in this study, identical to figure 2.
