# Supplementary material for: Butterfly dichromatism primarily evolved via Darwin's, not Wallace's, model
Source: Evol Lett. 2020 Oct 23;4(6):545–55. doi: 10.1002/evl3.199 (PMC7719551; doi:10.1002/evl3.199)
Supplement: Supplementary file 1 — Tables S1. Centroids of males and females in Lab color space, and dichromatism (defined as the centroid distance) Figure S1. Comparison between the structure in color space using either photographs or drawings for a subset of 53 species. Figure S2. Male and female rates of butterfly color evolution. Figure S3. Comparison of the observed ratio between male and female evolutionary rate. Figure S4. Coloration by sex of European butterflies, on the ventral sides. Figure S5. Male and female color evolution are strongly correlated, also on the ventral sides. Figure S6. Comparison of the observed ratio between male and female evolutionary rate with the expected distribution from permuted phenotypes. Figure S7. As ventral side dichromatism increases color evolution becomes evenly balanced between males and females. Figure S8. Changes in ventral side dichromatism are more likely to be the result of male change than female change. Figure S9. The phylogenetic tree as used in this study, identical to figure 2. [file EVL3-4-545-s001.docx]

Supplementary material

Table of contents:

[1.1 Methods 2](#_Toc47076943)

[1.2 Tables 4](#_Toc47076944)

[Tables S1: Centroids of males and females in Lab color space, and dichromatism (defined as the centroid distance). 4](#_Toc47076945)

[1.3 Figures 14](#_Toc47076946)

[Figure S1: Comparison between the structure in color space using either photographs or drawings for a subset of 53 species 14](#_Toc47076947)

[Figure S2: Male and female rates of butterfly color evolution 15](#_Toc47076948)

[Figure S3: Comparison of the observed ratio between male and female evolutionary rate 16](#_Toc47076949)

[Figure S4: Coloration by sex of European butterflies, on the ventral sides 17](#_Toc47076950)

[Figure S5. Male and female color evolution are strongly correlated, also on the ventral sides 18](#_Toc47076951)

[Figure S6: Comparison of the observed ratio between male and female evolutionary rate with the expected distribution from permuted phenotypes 19](#_Toc47076952)

[Figure S7: As ventral side dichromatism increases color evolution becomes evenly balanced between males and females 20](#_Toc47076953)

[Figure S8: Changes in ventral side dichromatism are more likely to be the result of male change than female change. 21](#_Toc47076954)

[Figure S9: The phylogenetic tree as used in this study, identical to figure 2 23](#_Toc47076955)

## Methods

As described in the main text, the dichromatism of a species was defined in our analysis as the distance between the male and female color centroid in LAB space. Given the large color diversity of butterflies, this simple metric performed well, and allowed detailed modelling of male and female phenotypes. However, we also evaluated the use of metrics that focused more on different aspects of the color distribution.

Firstly, we fitted alpha shapes around the complete color distributions in lab space for males and females separately. A dichromatism metric can then be derived by calculating what fraction of the total color volume (the union of male and female) is shared by both sexes (the intersection of male and female). In addition, one can calculate what fractions are uniquely male and uniquely female. This metric uses the full color spectrum, and closely follows the intuitive definition of color overlap.

Furthermore, we calculated a metric that used the average nearest neighbor distance, where for each observation (pixel) the nearest observation of the other sex was found. This approach has similarities to Dale *et al* 2015.

Finally, we attempted an approach based on the k-means color palette as used in figure 2, where a chi-squared statistic was calculated between the sexes. This approach aimed to limit the influence of very small patches, as well as the influence of consistent but slight differences in color. While these methods haves varying strengths of intuitive appeal, several problems became apparent with their use. For example, a species with a vibrantly violet male and brown female, would be assigned very low dichromatism (as per the alpha-shape method) if the female had a few very small violet spots. Conversely, a species with very similarly looking sexes may be assigned a high dichromatism score, if a very small marking used a unique color, or if large patches used a very subtly different color. In the end, our alternative dichromatism scores did not match the expectation of the human observers. The color centroid metric, however, performed much better than expected. Smaller color patches have a proportionally reduced influence, and smaller color changes carry less weight, and calculated dichromatism scores agreed with human assessment. Consequently, we decided for the more parsimonious centroid-based metric, which also allowed us to conveniently model the male and female contribution in the evolutionary history of dichromatism.

We finalized the color metric before any downstream analysis took place.

## Tables

### Tables S1: Centroids of males and females in Lab color space, and dichromatism (defined as the centroid distance).

| Species | L males | L females | a males | a females | b males | b females | dichromatism |
| --- | --- | --- | --- | --- | --- | --- | --- |
| Aglais urticae | 35.47 | 35.59 | 8.69 | 8.77 | 28.72 | 28.91 | 0.24 |
| Agriades glandon glandon | 59.24 | 39.61 | -2.76 | 1.02 | 17.83 | 21.71 | 20.37 |
| Agrodiaetus admetus | 26.72 | 31.6 | 11.75 | 14.24 | 20.77 | 25.67 | 7.34 |
| Agrodiaetus agenjoi | 33.04 | 42.62 | 7.59 | 2.21 | 28.7 | 29.6 | 11.03 |
| Agrodiaetus ainsae | 80.86 | 44.12 | -2.8 | 4.97 | 19.45 | 33.36 | 40.04 |
| Agrodiaetus amanda | 45.64 | 24.43 | 5.76 | 10.76 | -20.38 | 14.52 | 41.15 |
| Agrodiaetus aroaniensis | 27.72 | 32.41 | 11.2 | 9.84 | 22.01 | 23.82 | 5.2 |
| Agrodiaetus damon | 60.02 | 29.83 | -7.09 | 1.11 | 3.57 | 21.48 | 36.04 |
| Agrodiaetus dolus dolus | 71.03 | 36.92 | -2.8 | 5.29 | 12.92 | 26.68 | 37.66 |
| Agrodiaetus escheri | 49.93 | 33.4 | 10.52 | 5.93 | -28.51 | 24 | 55.24 |
| Agrodiaetus fabressei | 32.03 | 42.47 | 7.29 | 5.78 | 28.45 | 35.38 | 12.62 |
| Agrodiaetus galloi | 31.76 | 32.03 | 5.97 | 5.19 | 22.94 | 21.67 | 1.52 |
| Agrodiaetus humedasae | 18.25 | 20.44 | 6.06 | 5.7 | 14.33 | 16.59 | 3.17 |
| Agrodiaetus iphigenia | 52.78 | 29.61 | -0.46 | 1.43 | -23.27 | 18.5 | 47.8 |
| Agrodiaetus nephohiptamenos | 26.05 | 30.77 | 12.08 | 11.95 | 17.55 | 20.04 | 5.34 |
| Agrodiaetus pyrenaicus pyrenaicus | 66.31 | 37.32 | 0.55 | 0.7 | 13.63 | 11 | 29.11 |
| Agrodiaetus ripartii ripartii | 25.52 | 29.97 | 10.21 | 11.03 | 17.16 | 20.62 | 5.69 |
| Agrodiaetus thersites | 43.81 | 27.19 | 17.95 | 10.59 | -36.02 | 18.61 | 57.58 |
| Agrodiaetus violeta | 29.05 | 37.55 | 7.32 | 6.89 | 22.69 | 28.27 | 10.18 |
| Albulina orbitulus | 34.97 | 30.66 | 17.56 | 8.17 | -37.01 | 14.05 | 52.1 |
| Anthocharis belia euphenoides | 70.62 | 81.71 | -1.9 | -4.81 | 67.94 | 30.43 | 39.22 |
| Anthocharis cardamines | 74.74 | 79.23 | 3.08 | -5.78 | 23.65 | 11.76 | 15.49 |
| Anthocharis damone | 70.28 | 77.45 | 0.87 | -5.06 | 69.01 | 30.57 | 39.56 |
| Anthocharis gruneri | 63.83 | 75.62 | 3.66 | -4.06 | 46.83 | 21.51 | 28.97 |
| Apatura ilia ilia | 31.55 | 36.19 | 4.07 | -1.09 | 4.95 | 17.8 | 14.6 |
| Apatura iris | 30.83 | 36.35 | 1.51 | -1.3 | 6.71 | 18.54 | 13.35 |
| Apatura metis | 40.48 | 50.84 | 11.26 | 9.55 | 28.5 | 43.42 | 18.24 |
| Aphantopus hyperantus | 13.39 | 21.88 | 1.11 | 4.51 | 7.28 | 15.77 | 12.48 |
| Aporia crataegi | 83.31 | 83.14 | -4.02 | -3.04 | 13.61 | 12.41 | 1.56 |
| Araschnia levana | 37.44 | 39.31 | 11.21 | 10.85 | 30.24 | 32.08 | 2.65 |
| Archon apollinus | 64.22 | 46.17 | -4.51 | -3.07 | 27.64 | 24.05 | 18.46 |
| Arethusana arethusa arethusa | 52.01 | 42.94 | 7.85 | 8.87 | 30.3 | 30.82 | 9.14 |
| Argynnis adippe adippe | 40.56 | 46.36 | 12.42 | 5.14 | 40.93 | 42.88 | 9.51 |
| Argynnis aglaja aglaja | 36.81 | 36.85 | 13.06 | 6.87 | 36 | 32.93 | 6.9 |
| Argynnis elisa | 44.54 | 42.29 | 22.27 | 17.45 | 44.18 | 41.39 | 6.01 |
| Argynnis laodice | 40.52 | 41.69 | 17.39 | 13.4 | 39.36 | 39.89 | 4.2 |
| Argynnis niobe | 43.8 | 44.66 | 8.67 | 0.94 | 41.11 | 37.99 | 8.39 |
| Argynnis pandora | 35.38 | 38.57 | -2.41 | -3.05 | 27.63 | 29.21 | 3.62 |
| Argynnis paphia | 39.38 | 33.1 | 21.11 | 11.02 | 39.61 | 31.71 | 14.27 |
| Aricia agestis | 29.64 | 30.92 | 10.43 | 11.62 | 16.29 | 18.43 | 2.76 |
| Aricia artaxerxes | 23.97 | 26.79 | 7.72 | 9.32 | 16.9 | 19.74 | 4.31 |
| Aricia morronensis | 31.79 | 31.15 | 3.69 | 4.93 | 20.37 | 21.27 | 1.66 |
| Artogeia bryoniae | 79.66 | 60.93 | -7 | -5 | 12.48 | 43.17 | 36.01 |
| Artogeia ergane | 88.34 | 83.45 | -6.32 | -7.39 | 13.89 | 22.32 | 9.81 |
| Artogeia krueperi | 80.73 | 78.47 | -5.63 | -5.33 | 14.44 | 12.43 | 3.04 |
| Artogeia mannii | 85.76 | 84.53 | -4.25 | -3.56 | 11.06 | 10.85 | 1.43 |
| Artogeia napi | 78.18 | 78.12 | -3.97 | -3.98 | 18.33 | 18.24 | 0.1 |
| Artogeia napi napi | 79.11 | 78.87 | -5.05 | -4.98 | 15.38 | 15.31 | 0.26 |
| Artogeia napi segonzaci | 70.41 | 70.37 | -5.19 | -5.19 | 14.78 | 14.8 | 0.05 |
| Artogeia rapae | 84.99 | 82.23 | -5.94 | -6.17 | 16.45 | 25.93 | 9.87 |
| Azanus jesous | 60.29 | 51.75 | 7.78 | 3.03 | 12.76 | 34.11 | 23.48 |
| Azanus ubaldus | 67.92 | 61.23 | 2.94 | 4.9 | 21.76 | 40.91 | 20.38 |
| Berberia abdelkader | 30.11 | 30.16 | 5.58 | 5.6 | 21.17 | 21.12 | 0.07 |
| Berberia lambessanus | 27.25 | 27.2 | 3.68 | 3.69 | 16.47 | 16.38 | 0.1 |
| Boloria aquilonaris | 35.66 | 38.33 | 20.13 | 14.43 | 33.35 | 35.14 | 6.54 |
| Boloria graeca | 43.48 | 45.2 | 11.39 | 12.38 | 40.25 | 41.34 | 2.26 |
| Boloria napaea | 39.54 | 40.43 | 19.17 | 5.59 | 36.18 | 28.97 | 15.4 |
| Boloria pales pales | 41.18 | 37.85 | 18.91 | 19.55 | 37.11 | 33.73 | 4.79 |
| Brenthis daphne | 41.72 | 42.59 | 19.6 | 18.84 | 40.86 | 41.05 | 1.17 |
| Brenthis hecate | 38.02 | 41.03 | 19.99 | 12 | 35.71 | 37.86 | 8.81 |
| Brenthis ino | 44.39 | 35.59 | 19.59 | 10.15 | 41.81 | 32.67 | 15.81 |
| Cacyreus marshalli | 33.6 | 31.65 | -3.75 | -5.49 | 26.67 | 25.99 | 2.7 |
| Callophrys avis | 29.37 | 36.83 | 10.66 | 13.3 | 16.34 | 24.2 | 11.15 |
| Callophrys rubi | 30.98 | 34.42 | 6.3 | 6.57 | 12.97 | 14.79 | 3.9 |
| Catopsila florella | 88.21 | 87.98 | -7.29 | -10.92 | 15.91 | 63.5 | 47.73 |
| Celastrina argiolus | 49.76 | 48.85 | 11.14 | 8.32 | -33.69 | -25.22 | 8.98 |
| Charaxes jasius | 26.8 | 26.68 | 7.46 | 7.35 | 15.9 | 15.83 | 0.18 |
| Chazara briseis | 33.7 | 44.9 | 4.41 | 5.2 | 20.72 | 21.87 | 11.28 |
| Chazara prieuri | 51.47 | 49.26 | -0.26 | 0.68 | 14.22 | 22.03 | 8.18 |
| Chilades trochylus | 33.81 | 34.56 | -0.88 | 1.23 | 19.97 | 21.38 | 2.65 |
| Cigaritis allardi | 36.52 | 40.07 | 16.28 | 15.04 | 28.67 | 33.48 | 6.11 |
| Cigaritis siphax | 46.99 | 46.68 | 19.83 | 17.07 | 39.81 | 39.19 | 2.85 |
| Cigaritis zohra | 40.4 | 51.97 | 14.14 | 9.82 | 32.43 | 34.84 | 12.59 |
| Clossiana chariclea | 31.02 | 36 | 11.21 | 12.05 | 28.1 | 34.32 | 8.01 |
| Clossiana dia | 32.37 | 36.31 | 13.61 | 11.43 | 31.21 | 35.38 | 6.14 |
| Clossiana euphorysne | 38.49 | 37.41 | 16.71 | 14.79 | 36.74 | 35.69 | 2.44 |
| Clossiana freija | 34.04 | 36.65 | 9.78 | 3.14 | 31.88 | 32.62 | 7.17 |
| Clossiana frigga | 30.26 | 27.65 | 12.85 | 7.89 | 28.44 | 26.32 | 5.99 |
| Clossiana improba | 32.71 | 36.65 | 1.16 | 5.32 | 19.3 | 26.57 | 9.25 |
| Clossiana polaris | 38.74 | 36.42 | 6.68 | 3.49 | 34.86 | 33.19 | 4.28 |
| Clossiana selene | 32.87 | 33.9 | 13.39 | 7.9 | 31.87 | 29.67 | 6.01 |
| Clossiana thore thore | 25.05 | 28.06 | 6.04 | 9.03 | 21.38 | 25.85 | 6.16 |
| Clossiana titania titania | 38.89 | 44 | 10.19 | 7.44 | 38.94 | 42.62 | 6.88 |
| Coenonympha arcania | 40.49 | 46.16 | 14.86 | 16.88 | 26.23 | 30.77 | 7.54 |
| Coenonympha arcanioides | 39.18 | 41.54 | 15.63 | 15.98 | 29.54 | 32.06 | 3.47 |
| Coenonympha corinna | 51.52 | 55.22 | 18.05 | 18.02 | 36.92 | 38.05 | 3.87 |
| Coenonympha darwiniana | 37.06 | 38.79 | 15.5 | 13.84 | 24.45 | 24.39 | 2.39 |
| Coenonympha dorus dorus | 44.08 | 55.27 | 10.71 | 14.27 | 35.85 | 44.74 | 14.73 |
| Coenonympha elbana | 50.47 | 54.12 | 19.73 | 17.72 | 33.96 | 35.68 | 4.5 |
| Coenonympha gardetta | 39.11 | 48.79 | 9.94 | 9.27 | 22.52 | 27.85 | 11.07 |
| Coenonympha glycerion glycerion | 39.65 | 55.88 | 8.22 | 7.74 | 29.93 | 41.73 | 20.07 |
| Coenonympha hero | 33.26 | 41.53 | 1.12 | 2.96 | 18.55 | 25.59 | 11.01 |
| Coenonympha leander leander | 38.99 | 49.3 | 8.91 | 9.34 | 29.98 | 39.07 | 13.74 |
| Coenonympha oedippus | 19.47 | 29.63 | 9.03 | 7.85 | 8.71 | 12.33 | 10.85 |
| Coenonympha pamphilus | 61.48 | 71.63 | 12.25 | 6.06 | 35.89 | 37.41 | 11.99 |
| Coenonympha rhodopensis | 56.16 | 60.96 | 10.81 | 11.57 | 41.45 | 46.69 | 7.15 |
| Coenonympha thyrsis | 62.34 | 70.65 | 12.69 | 8.33 | 42.41 | 42.71 | 9.39 |
| Coenonympha tullia tullia | 57.2 | 63.02 | 7.01 | 8.21 | 33.52 | 35.22 | 6.18 |
| Coenonympha vaucheri | 49.39 | 59.88 | 12.43 | 9.2 | 37.24 | 43.1 | 12.44 |
| Colias alfacariensis | 77 | 74.62 | -11.91 | -6.16 | 55.94 | 14.35 | 42.04 |
| Colias aurorina | 48.99 | 50.18 | 14.88 | 7.5 | 37.04 | 41.83 | 8.89 |
| Colias caucasia | 51.04 | 51.22 | 16.7 | 7.38 | 41.1 | 37.58 | 9.96 |
| Colias chrysotheme | 56.91 | 56.88 | 4.07 | 4.13 | 45.91 | 45.83 | 0.11 |
| Colias crocea | 55.54 | 49.19 | 7.32 | 11.03 | 49.07 | 41.09 | 10.85 |
| Colias crocea helice | 59.37 | 59.25 | -2.05 | -2.02 | 14.78 | 14.65 | 0.19 |
| Colias erate | 67.34 | 63.34 | -7.2 | -3.85 | 53.66 | 49.12 | 6.91 |
| Colias erate white form | 66.09 | 66.12 | -0.77 | -0.77 | 10.12 | 10.12 | 0.03 |
| Colias hecla | 57.94 | 54.69 | 15.52 | 10.01 | 46.23 | 39.88 | 9.02 |
| Colias hyale | 72.55 | 71.93 | -9.69 | -5.49 | 51.84 | 20.27 | 31.86 |
| Colias myrmidone | 55.53 | 57.3 | 12.91 | 8.62 | 46.43 | 48.77 | 5.2 |
| Colias nastes | 68.62 | 63.26 | -8.32 | -4.08 | 23.8 | 16.01 | 10.37 |
| Colias palaeno | 67.46 | 72.5 | -9.32 | -2.81 | 33 | 11.66 | 22.88 |
| Colias phicomone | 57.87 | 67.54 | -6.05 | -2.16 | 31.37 | 14.19 | 20.09 |
| Colotis evagore | 77.02 | 68.69 | 1.88 | 1.27 | 11.88 | 13.88 | 8.59 |
| Cupido carswelli | 26.2 | 29.69 | -5.43 | -5.6 | 12.03 | 18.92 | 7.72 |
| Cupido lorquinii | 33.07 | 27.78 | 10.84 | -7.69 | -24.42 | 14.83 | 43.73 |
| Cupido minimus | 28.44 | 27.55 | -1.34 | -2.78 | 17.37 | 22.15 | 5.07 |
| Cupido osiris | 37.91 | 31.43 | 17.47 | -6.74 | -37.04 | 20.12 | 62.42 |
| Cyaniris semiargus semiargus | 26.47 | 22.76 | 23.52 | 7.2 | -33.38 | 10.37 | 46.83 |
| Cyclyrius webbianus | 30.53 | 25.33 | 13.85 | 15.65 | 1.75 | 18.92 | 18.03 |
| Danaus chrysippus | 42.07 | 42.04 | 19.09 | 18.98 | 37.33 | 37.17 | 0.2 |
| Danaus plexippus | 34.51 | 33.6 | 19.44 | 20.71 | 31.89 | 26.06 | 6.03 |
| Elphinstonia charlonia | 70.33 | 70.63 | -19.2 | -19.22 | 63.83 | 64.02 | 0.35 |
| Elphinstonia penia | 74 | 73.92 | -14.56 | -14.55 | 67.2 | 67.3 | 0.12 |
| Erebia aethiopella | 21.12 | 26.83 | 10.91 | 11.42 | 14.07 | 19.2 | 7.69 |
| Erebia aethiops | 9.11 | 16.99 | 1.94 | 4.14 | 6.15 | 15.03 | 12.07 |
| Erebia alberganus | 20.03 | 23.33 | 4.59 | 4.39 | 14.88 | 17.43 | 4.17 |
| Erebia calcaria | 24.86 | 32.46 | 6.52 | 9.25 | 20.6 | 26.8 | 10.18 |
| Erebia cassioides | 15.16 | 22.27 | 2.97 | 3.54 | 11.84 | 16.99 | 8.8 |
| Erebia christi | 27.28 | 41.45 | 7.6 | 9.66 | 20.68 | 31.36 | 17.87 |
| Erebia claudina | 28.17 | 34.71 | 11.88 | 11.22 | 25.49 | 28.6 | 7.27 |
| Erebia disa | 30.48 | 39.37 | 6.19 | 4.95 | 20.73 | 25.06 | 9.97 |
| Erebia embla | 31.45 | 35.37 | 6.26 | 6.12 | 22.81 | 25.09 | 4.54 |
| Erebia epiphron silesiana | 28.63 | 37.05 | 7.55 | 7.51 | 24.64 | 29.62 | 9.77 |
| Erebia epistygne | 31.05 | 37.38 | 6.92 | 7.44 | 26.14 | 29.51 | 7.19 |
| Erebia eriphyle | 21.69 | 23.31 | 5.94 | 4.96 | 18.33 | 18.35 | 1.89 |
| Erebia euryale euryale | 14.3 | 22.3 | 5.76 | 6.97 | 8.57 | 14.03 | 9.76 |
| Erebia flavofasciata | 32.25 | 36.16 | 9.47 | 9.36 | 26.49 | 28.91 | 4.59 |
| Erebia gorge | 23.97 | 30.84 | 11.38 | 9.98 | 16.08 | 19.07 | 7.63 |
| Erebia gorgone | 21.49 | 31.54 | 4.89 | 8.01 | 16.8 | 24.93 | 13.29 |
| Erebia hispania | 21.08 | 30.58 | 5.88 | 6.2 | 18.57 | 27.72 | 13.19 |
| Erebia lefebvrei lefebvrei | 11.39 | 21.7 | 5.26 | 11.44 | 4.29 | 13.8 | 15.33 |
| Erebia ligea | 30.19 | 24.92 | 10.74 | 16.1 | 15 | 23.08 | 11.04 |
| Erebia manto manto | 26.88 | 34.7 | 3.64 | 4.68 | 21.06 | 26.34 | 9.49 |
| Erebia medusa medusa | 22.67 | 27.09 | 10.47 | 11.25 | 21.01 | 24.56 | 5.72 |
| Erebia melampus | 27.95 | 38.65 | 5.73 | 6.58 | 18.64 | 25.39 | 12.69 |
| Erebia melas schanerdae | 10.8 | 15.84 | 2.4 | 4.99 | 8.26 | 13.9 | 7.99 |
| Erebia meolans meolans | 17.49 | 27.3 | 8.11 | 10.41 | 13.37 | 21.57 | 12.99 |
| Erebia mnestra | 31.52 | 40.34 | 7.04 | 6.21 | 20.67 | 23.84 | 9.41 |
| Erebia montana montana | 20.88 | 24.42 | 12.75 | 13.96 | 12.1 | 15.17 | 4.84 |
| Erebia neoridas | 22.65 | 24.84 | 13.42 | 10.91 | 16.62 | 17.3 | 3.4 |
| Erebia nivalis | 24.59 | 29.09 | 8.44 | 7.83 | 21.36 | 24.96 | 5.79 |
| Erebia oeme oeme | 14.37 | 21.18 | 7.07 | 8.53 | 10.49 | 14.96 | 8.27 |
| Erebia orientalis | 23.16 | 31.63 | 4.54 | 5.17 | 17.09 | 21.85 | 9.74 |
| Erebia ottomana | 29.91 | 33.66 | 5.63 | 7.59 | 17.35 | 22.16 | 6.4 |
| Erebia palarica | 21.57 | 27.38 | 15.22 | 14.39 | 7.93 | 10.98 | 6.62 |
| Erebia pandrose | 25.29 | 32.24 | 11.89 | 13.87 | 11 | 14.53 | 8.04 |
| Erebia pharte pharte | 19.21 | 27.86 | 2.62 | 3.03 | 10.25 | 16.01 | 10.4 |
| Erebia pluto pluto | 17.05 | 29.06 | 3.51 | 6.61 | 9.39 | 19.26 | 15.85 |
| Erebia polaris | 20.08 | 26.23 | 3.92 | 1.97 | 14.1 | 17.05 | 7.09 |
| Erebia pronoe pronoe | 17.15 | 23.27 | 6.83 | 8.02 | 12.89 | 18.18 | 8.18 |
| Erebia rhodopensis | 29.53 | 38.92 | 6.59 | 8.95 | 20.12 | 26.16 | 11.41 |
| Erebia scipio | 19.94 | 26.94 | 12.25 | 13.42 | 14.51 | 20.11 | 9.05 |
| Erebia sthennyo | 20.35 | 34.33 | 12.12 | 12.9 | 7.44 | 14.66 | 15.75 |
| Erebia stirius | 16.42 | 26.13 | 5.31 | 9.66 | 7.14 | 16.13 | 13.93 |
| Erebia styx styx | 18.24 | 20.23 | 5.46 | 5.5 | 14.11 | 15.26 | 2.31 |
| Erebia sudetica sudetica | 23.56 | 32.31 | 5.86 | 7.56 | 15.37 | 22.87 | 11.65 |
| Erebia triaria triaria | 27.74 | 35.38 | 3.85 | 7.99 | 18.69 | 25.77 | 11.21 |
| Erebia tyndarus | 20.82 | 32.21 | 3.66 | 6.09 | 17.25 | 25.7 | 14.39 |
| Erebia zapateri | 17.24 | 25.26 | 10.94 | 9.31 | 10.71 | 15.21 | 9.34 |
| Euchloe ausonia | 75.08 | 75.89 | -3.21 | -4.63 | 9.67 | 18.51 | 8.99 |
| Euchloe belemia | 74.83 | 79.21 | -2.1 | -1.97 | 8.1 | 7.93 | 4.39 |
| Euchloe falloui | 79.68 | 76.37 | -1.1 | -2.53 | 6.4 | 14.75 | 9.09 |
| Euchloe insolaris | 75.71 | 80.16 | -3.56 | -4.53 | 10.41 | 9.68 | 4.61 |
| Euchloe simplonia | 73.53 | 74.37 | -4.16 | -2.31 | 13.34 | 19.6 | 6.58 |
| Euchloe tagis tagis | 78.21 | 78.35 | -1.84 | -1.84 | 11.77 | 11.78 | 0.15 |
| Eumedonia eumedon | 17.36 | 26.91 | -2.46 | -1.8 | 9.28 | 19.21 | 13.8 |
| Eurodryas aurinia | 38.08 | 40.32 | 5.38 | 3.7 | 29.71 | 31.17 | 3.16 |
| Eurodryas desfontainii | 42.46 | 44 | 8.18 | 5.88 | 34.45 | 34.72 | 2.79 |
| Everes alcetas | 50.1 | 29.48 | 12.4 | 3.6 | -24.89 | 18.74 | 49.06 |
| Everes argiades | 47.33 | 45.04 | 10.46 | 3.73 | -23.15 | 6.48 | 30.46 |
| Everes decoloratus | 50.81 | 24.13 | 2.04 | 4.03 | -10.78 | 14.16 | 36.57 |
| Glaucopsyche alexis | 47.3 | 32.57 | 6.65 | -1.29 | -29.76 | 21.76 | 54.18 |
| Glaucopsyche melanops | 56.11 | 48.69 | 6.79 | 0.84 | -24.21 | -3.05 | 23.2 |
| Gonepteryx cleopatra | 84.68 | 91.75 | -6.74 | -9.54 | 74.38 | 33.92 | 41.17 |
| Gonepteryx farinosa | 89.81 | 92.8 | -15.1 | -5.65 | 71.04 | 16.85 | 55.09 |
| Gonepteryx rhamni | 88 | 91.46 | -15.49 | -6.36 | 72.78 | 17.39 | 56.24 |
| Hamearis lucina | 18.5 | 26.07 | 7.79 | 10.18 | 14.01 | 22.65 | 11.73 |
| Hipparchia alcyone | 33.49 | 31.09 | 5.7 | 1.34 | 16.44 | 16.68 | 4.98 |
| Hipparchia aristaeus aristaeus | 34.84 | 40.37 | 9.94 | 11.66 | 28.71 | 32.77 | 7.07 |
| Hipparchia azorina | 29.31 | 44.58 | 5.97 | 3.51 | 19.16 | 23.12 | 15.97 |
| Hipparchia caldeirense | 29.98 | 44.85 | 4.72 | 3.17 | 19.9 | 24.08 | 15.53 |
| Hipparchia cretica | 41.57 | 35.75 | 5.73 | 6.07 | 32.03 | 28.43 | 6.85 |
| Hipparchia ellena | 32.51 | 35.04 | 0.2 | -0.36 | 10.73 | 11.31 | 2.66 |
| Hipparchia fagi | 37.89 | 34.29 | 3.75 | 2.56 | 15.76 | 12.78 | 4.82 |
| Hipparchia mersina | 43.38 | 43.21 | 4.62 | 4.63 | 26.12 | 26.12 | 0.17 |
| Hipparchia neomiris | 39.1 | 40.8 | 2.86 | 4.85 | 23.21 | 31.8 | 8.98 |
| Hipparchia pellucida | 36.35 | 38.09 | 0.6 | -1.05 | 26.06 | 23.65 | 3.4 |
| Hipparchia semele | 29.23 | 23.81 | 4.54 | 4.27 | 21.1 | 17.46 | 6.53 |
| Hipparchia syriaca | 30.68 | 37.66 | 2.2 | 1.96 | 17.88 | 18.89 | 7.05 |
| Hipparchia volgenis | 33.86 | 42.02 | 4.36 | 4.93 | 21.19 | 28.47 | 10.95 |
| Hypodryas cynthia | 34.19 | 36.27 | -1.18 | 5.43 | 13.82 | 30.8 | 18.34 |
| Hypodryas iduna | 35.22 | 40.89 | -1.56 | -0.12 | 20.17 | 24.52 | 7.29 |
| Hypodryas intermedia | 42.46 | 38.07 | 3.68 | 5.33 | 29.67 | 28.32 | 4.88 |
| Hypodryas maturna | 27.86 | 31.32 | 10.66 | 13.5 | 21.59 | 26.27 | 6.47 |
| Hyponephele lupina | 32.98 | 39.3 | 1.43 | 2.05 | 23.63 | 26.42 | 6.93 |
| Hyponephele lycaon | 50.76 | 45.2 | 9.04 | 9.35 | 22.35 | 25.84 | 6.57 |
| Hyponephele maroccana | 36.08 | 51.35 | 8.94 | 7.84 | 21.98 | 24.49 | 15.52 |
| Inachis io | 32.04 | 32 | 6.17 | 6.28 | 21.77 | 21.65 | 0.17 |
| Iolana iolas | 50.46 | 41.96 | 13.48 | 8.38 | -24.3 | 9.89 | 35.6 |
| Iphiclides podalirius | 50.05 | 49.97 | -4.92 | -4.92 | 25.43 | 25.24 | 0.2 |
| Issoria lathonia | 42.41 | 41.02 | 16.74 | 9.15 | 38.83 | 35.66 | 8.34 |
| Kanetisa circe | 36.3 | 34.46 | 2.51 | 3.44 | 19.34 | 14.54 | 5.23 |
| Kirinia climene | 45.55 | 45.32 | 9.11 | 9.06 | 34.76 | 34.56 | 0.31 |
| Kirinia roxelana | 38.39 | 45.08 | 10.35 | 9.69 | 31.6 | 34.09 | 7.17 |
| Kretania eurypilus | 37.51 | 39.44 | 9.13 | 11.46 | 21.75 | 23.27 | 3.38 |
| Kretania psylorita | 37.1 | 43.64 | 9.95 | 11.61 | 20.81 | 29.07 | 10.66 |
| Laeopsis roboris | 27.74 | 27.76 | 8.12 | 5.51 | -0.06 | 3.53 | 4.44 |
| Lampides boeticus | 49.63 | 39.74 | 2.16 | -6.1 | 9.8 | 22 | 17.74 |
| Lasiommata maera maera | 39.94 | 49.07 | 11.13 | 12.87 | 28.19 | 33.14 | 10.53 |
| Lasiommata megera | 41.29 | 39.89 | 6.66 | 10.37 | 27.27 | 34.58 | 8.32 |
| Lasiommata petropolitana | 33.24 | 32.25 | 6.44 | 8.23 | 22.35 | 26.85 | 4.94 |
| Leptidea duponcheli | 82.1 | 85.72 | -3.73 | -4.42 | 13.47 | 12.3 | 3.86 |
| Leptidea morsei | 87.95 | 86.9 | -0.6 | -0.59 | 0.81 | 1.1 | 1.09 |
| Leptidea sinapis | 86.34 | 86.74 | -1.1 | -1.31 | 2.19 | 4.15 | 2 |
| Leptotes pirithous | 50.81 | 49.47 | 3.23 | -2.39 | 11.21 | 29.82 | 19.48 |
| Libythea celtis | 23.07 | 23.03 | 13.03 | 13.02 | 22.21 | 22.17 | 0.06 |
| Limenitis camilla | 22.49 | 22.62 | 0.22 | 0.21 | 7.66 | 7.7 | 0.14 |
| Limenitis populi | 25.29 | 31.48 | 1.8 | 2.54 | 9.02 | 13.83 | 7.88 |
| Limenitis reducta | 23.68 | 23.62 | -0.41 | -0.35 | -1.11 | -1.14 | 0.09 |
| Lopinga achine | 33.41 | 41.54 | 4.81 | 10.23 | 29.25 | 27.55 | 9.92 |
| Lycaena alciphron alciphron | 43.32 | 24.04 | 13.23 | 2.85 | 31.31 | 19.51 | 24.88 |
| Lycaena candens | 33.81 | 32.64 | 21.72 | 10.41 | 30.27 | 29.45 | 11.4 |
| Lycaena dispar | 40.88 | 26.65 | 30.6 | 16.1 | 37.61 | 20.87 | 26.33 |
| Lycaena helle | 22.31 | 22.69 | 16.18 | 12.6 | -1.01 | 10.56 | 12.11 |
| Lycaena hippothoe hippothoe | 39.05 | 35.65 | 30.4 | 14.82 | 35.55 | 30.3 | 16.78 |
| Lycaena ottomana | 42.94 | 38.69 | 33.64 | 19.6 | 43.62 | 36.24 | 16.42 |
| Lycaena phlaeas | 29 | 27.29 | 14.05 | 12.89 | 20.84 | 15.86 | 5.39 |
| Lycaena phoebus | 45.13 | 45.87 | 32.27 | 26.09 | 42.25 | 43.5 | 6.34 |
| Lycaena thersamon | 46.76 | 40.98 | 31.23 | 22.13 | 43.03 | 36.05 | 12.85 |
| Lycaena thetis | 49.33 | 55.09 | 32.26 | 10.35 | 46.8 | 46.97 | 22.66 |
| Lycaena tityrus tityrus | 25.3 | 27.42 | -0.86 | 8.32 | 13.15 | 20.11 | 11.71 |
| Lycaena virgaureae | 45.91 | 34.65 | 34.4 | 17.54 | 46.8 | 32.18 | 25 |
| Lysandra albicans | 78.92 | 29.42 | -1.41 | 9.27 | 7.68 | 16.82 | 51.47 |
| Lysandra bellargus | 50.45 | 22.03 | 9.2 | 8.1 | -30.7 | 11.1 | 50.56 |
| Lysandra coridon | 67.68 | 29.11 | -5.83 | 2.63 | 3.29 | 17.77 | 42.06 |
| Lysandra hispana | 66.48 | 22.54 | 0.83 | 10.06 | -2.25 | 11.81 | 47.05 |
| Lysandra philippi | 60.43 | 28.04 | -1.05 | 7.55 | -4.3 | 14.5 | 38.42 |
| Lysandra punctifera | 56.92 | 27.28 | 5.54 | 12.8 | -22.94 | 18.07 | 51.13 |
| Maculinea alcon | 47.38 | 38.27 | 12.07 | -2.01 | -18.49 | 22.88 | 44.64 |
| Maculinea arion arion | 49.49 | 47.97 | 9.95 | 6.07 | -14.46 | -4.32 | 10.96 |
| Maculinea nausithous | 39.03 | 34.39 | 1.7 | -0.31 | 15.95 | 30.64 | 15.53 |
| Maculinea rebeli | 51.99 | 47.06 | 12.06 | 3.05 | -21.78 | 10.06 | 33.46 |
| Maculinea telejus | 56.16 | 43.73 | 0.78 | 0.16 | -2.71 | 12.79 | 19.88 |
| Maniola jurtina jurtina | 22.56 | 35.02 | 2.79 | 5.28 | 17.09 | 27.42 | 16.38 |
| Maniola nurag | 39.79 | 49.02 | 9.39 | 13.69 | 31.45 | 40.08 | 13.35 |
| Maniola telmessia | 30.12 | 35.83 | 11.11 | 14.12 | 17.67 | 24.42 | 9.33 |
| Maurus vogelii | 43.25 | 39.93 | 6.33 | 6.87 | 26.68 | 26.88 | 3.37 |
| Melanargia arge | 68.56 | 64.96 | -3.34 | -3.03 | 13.59 | 7.51 | 7.07 |
| Melanargia galathea | 45.57 | 52.81 | -6.31 | -6.68 | 14.27 | 13.85 | 7.26 |
| Melanargia ines | 50.15 | 56.29 | -1.12 | -1.49 | 0.61 | 7.77 | 9.44 |
| Melanargia lachesis | 60.5 | 71.69 | -2.87 | -2.32 | 3.82 | 3.77 | 11.21 |
| Melanargia larissa | 57.3 | 57.52 | -1.56 | -1.64 | 17.25 | 17.18 | 0.25 |
| Melanargia occitanica occitanica | 53.97 | 60.31 | -4.33 | -2.46 | 9.5 | 9.36 | 6.61 |
| Melanargia russiae cleanthe | 53.44 | 56.16 | -5.19 | -4.38 | 13.67 | 16.23 | 3.82 |
| Meleageria daphnis | 55.11 | 42.26 | -4.05 | 5.72 | -12.33 | -10.36 | 16.26 |
| Melitaea aetherica | 37.35 | 34.08 | 22.12 | 10.55 | 37.4 | 31.69 | 13.31 |
| Melitaea arduinna | 39.24 | 34.03 | 11.73 | -0.43 | 35.81 | 29.89 | 14.49 |
| Melitaea cinxia | 37.7 | 34.7 | 9.1 | -0.18 | 32.72 | 27.06 | 11.28 |
| Melitaea deserticola | 44.34 | 51.88 | 22.51 | 16.93 | 44.41 | 51.4 | 11.69 |
| Melitaea diamina diamina | 27.37 | 34.19 | 8.36 | 6.38 | 24.65 | 29.37 | 8.53 |
| Melitaea didyma didyma | 41.18 | 45.13 | 15.09 | 11.4 | 40.87 | 43.89 | 6.2 |
| Melitaea phoebe phoebe | 34.26 | 37.34 | 3.22 | 2.38 | 28.48 | 33.2 | 5.69 |
| Melitaea trivia trivia | 37.78 | 36.55 | 12.29 | 11.06 | 35.02 | 35.46 | 1.8 |
| Mellicta asteria | 35.11 | 39.94 | 3.52 | 2.33 | 21.62 | 24.19 | 5.6 |
| Mellicta athalia athalia | 24.72 | 30.38 | 10.22 | 10.71 | 23.02 | 27.06 | 6.97 |
| Mellicta aurelia | 29.49 | 37.17 | 11.23 | 9.12 | 25.6 | 29.81 | 9.02 |
| Mellicta britomartis | 23.74 | 31.9 | 10.42 | 12.69 | 20.16 | 27.82 | 11.41 |
| Mellicta deione deione | 35.27 | 37.2 | 7.67 | -0.26 | 33.71 | 31.85 | 8.38 |
| Mellicta parthenoides | 39.8 | 41.83 | 13.22 | 9.21 | 35.84 | 36.76 | 4.59 |
| Mellicta varia | 32.75 | 34.93 | 17.37 | 11.51 | 28.44 | 28.43 | 6.25 |
| Minois dryas | 31.38 | 42.03 | 4.29 | 3.13 | 19.88 | 24.78 | 11.78 |
| Neohipparchia fatua | 28.18 | 34.58 | 5.58 | 7.03 | 16.3 | 23.24 | 9.56 |
| Neohipparchia hansii | 37.56 | 54.18 | 6.79 | 4.2 | 30.56 | 40.7 | 19.63 |
| Neohipparchia powelli | 41.56 | 51.78 | 3.14 | 2.5 | 30.34 | 33.85 | 10.82 |
| Neohipparchia statilinus statilinus | 31 | 39.35 | 4.41 | 7.67 | 16.81 | 30.68 | 16.52 |
| Neolysandra coelestina | 27.24 | 26.57 | 18.96 | 9.3 | -34.27 | 15.61 | 50.81 |
| Neptis rivularis | 25.13 | 25.14 | -1.35 | -1.35 | 8.11 | 8.1 | 0.01 |
| Neptis sappho | 36.09 | 36.3 | -1.23 | -1.27 | 10.31 | 10.26 | 0.22 |
| Nymphalis antiopa | 29.88 | 29.85 | -1.86 | -1.84 | 18.37 | 18.32 | 0.07 |
| Nymphalis polychlorus | 37.21 | 37.3 | 7.45 | 7.48 | 31.41 | 31.47 | 0.11 |
| Nymphalis vaualbum | 32.06 | 31.87 | 8.22 | 8.19 | 26.52 | 26.26 | 0.32 |
| Nymphalis xanthomelas | 34.71 | 34.84 | 12.25 | 12.4 | 29.22 | 29.4 | 0.26 |
| Oeneis bore | 51.5 | 51.54 | 6.28 | 7.86 | 38.61 | 42.62 | 4.31 |
| Oeneis glacialis | 47.74 | 51.79 | 5.94 | 7.14 | 34.76 | 41.67 | 8.1 |
| Oeneis jutta | 36.03 | 38.04 | 3.71 | 7.49 | 22.62 | 20.71 | 4.69 |
| Oeneis norna | 56.88 | 50.11 | 6.3 | 11.54 | 42.44 | 42.04 | 8.57 |
| Papilio alexanor | 50.04 | 50.05 | -6.8 | -6.74 | 35.38 | 35.36 | 0.07 |
| Papilio hospiton | 38.89 | 39.11 | -4.23 | -4.28 | 28.33 | 28.48 | 0.27 |
| Papilio machaon britannicus | 43.36 | 43.41 | -9.2 | -9.23 | 34.87 | 34.94 | 0.09 |
| Papilio machaon gorganus | 45.05 | 45.05 | -8.76 | -8.72 | 35.07 | 34.97 | 0.1 |
| Pararge aegeria aegeria | 32.2 | 37.16 | 11.64 | 10.67 | 21.4 | 23.74 | 5.57 |
| Pararge xiphia | 30.51 | 27.19 | 9.9 | 11.74 | 19.35 | 24.12 | 6.1 |
| Pararge xiphioides | 27.55 | 27.2 | 9.24 | 9.87 | 17.41 | 17.5 | 0.72 |
| Parnassius apollo | 66.44 | 66.42 | -2.45 | -2.46 | 10.47 | 10.52 | 0.05 |
| Parnassius apollo rhodopensis | 58.31 | 57.88 | -2.55 | -2.52 | 15.91 | 15.76 | 0.45 |
| Parnassius mnemosyne athene | 71.07 | 71.13 | -5.17 | -5.17 | 18.62 | 18.65 | 0.08 |
| Parnassius phoebus | 73.77 | 62.37 | -3.3 | -1.15 | 19.84 | 18.55 | 11.67 |
| Parnassius phoebus cardinalis | 59.36 | 59.48 | -2.64 | -2.73 | 28.28 | 28.34 | 0.16 |
| Pieris brassicae | 86.38 | 80.67 | -5.45 | -6.88 | 13.84 | 23.44 | 11.26 |
| Pieris cheiranthi | 72.86 | 72.78 | -4.58 | -4.61 | 22.47 | 22.46 | 0.09 |
| Plebejus allardi | 53.88 | 38.9 | 12.12 | 4.16 | -18.19 | 5.63 | 29.25 |
| Plebejus argus argus | 40.98 | 27.59 | 10.66 | -1.58 | -24.04 | 21.6 | 49.11 |
| Plebejus argyrognomon | 29.05 | 32.75 | 20.36 | 14.64 | -28.36 | -4.03 | 25.26 |
| Plebejus idas idas | 46.53 | 35.76 | 14.92 | 8.36 | -11.03 | 1.52 | 17.79 |
| Plebejus loewii | 49.44 | 33.84 | 2.48 | 3.26 | -26.26 | 26.17 | 54.71 |
| Plebejus martini | 52.55 | 45.73 | 13.01 | 4.48 | -19.44 | 9.19 | 30.64 |
| Plebejus pylaon sephirus | 29.72 | 29.71 | 10.25 | 10.23 | 19.59 | 19.53 | 0.07 |
| Plebicula atlantica | 57.67 | 30.08 | 6.71 | 13.64 | -19.73 | 16.97 | 46.43 |
| Plebicula dorylas | 55.33 | 32.05 | 4.54 | 9.53 | -29.91 | 19.64 | 54.97 |
| Plebicula golgus golgus | 54.32 | 37.03 | 0.9 | 9.93 | -25.06 | 22.5 | 51.41 |
| Plebicula nivescens | 75.69 | 31.07 | 2.42 | 12.62 | 4.13 | 17.21 | 47.61 |
| Polygonia c-album | 35.49 | 37.47 | 9.03 | 11.29 | 29.65 | 32.61 | 4.21 |
| Polygonia egea | 39.54 | 50.66 | 15.73 | 18.89 | 34.89 | 48.21 | 17.63 |
| Polyommatus eroides | 45.89 | 28.43 | 2.51 | 11.93 | -27.13 | 17.86 | 49.17 |
| Polyommatus eros | 61.63 | 37.31 | -6.62 | 9.22 | -6.42 | 19.83 | 39.14 |
| Polyommatus icarus icarus | 36.18 | 23.09 | 25.1 | 13.91 | -39.18 | 13.76 | 55.67 |
| Polyommatus menelaos | 65.79 | 45.24 | -6.37 | 7.02 | -1.32 | 24.84 | 35.85 |
| Pontia callidice | 80.3 | 67.88 | -3.25 | -1.46 | 11.37 | 12.54 | 12.61 |
| Pontia chloridice | 84.33 | 73.48 | -3.79 | -2.52 | 14.43 | 12.66 | 11.06 |
| Pontia daplidice | 74.08 | 65.52 | -5.58 | -5.28 | 17.58 | 15.58 | 8.79 |
| Proclossiana eunomia eunomia | 41.91 | 42.11 | 7.76 | 7.92 | 39.59 | 39.86 | 0.37 |
| Proterebia afra | 23.25 | 32.14 | 5.49 | 8.22 | 17.84 | 27.74 | 13.59 |
| Pseudaricia nicias | 53.21 | 35.23 | -3.37 | 8.7 | 19.82 | 25.84 | 22.47 |
| Pseudochazara anthelea amalthea | 34.43 | 34.36 | 5.42 | 5.46 | 25.82 | 25.81 | 0.08 |
| Pseudochazara anthelea anthelea | 34.26 | 42.63 | 2.72 | 8.92 | 20.98 | 35.39 | 17.78 |
| Pseudochazara atlantis | 53.48 | 57.79 | 4.77 | 3.12 | 35.16 | 42.43 | 8.61 |
| Pseudochazara cingovskii | 41.05 | 54.4 | 5.42 | 2.04 | 26.92 | 33.44 | 15.24 |
| Pseudochazara geyeri | 52.51 | 48.32 | 4.12 | 6.06 | 31.5 | 35.02 | 5.81 |
| Pseudochazara graeca graeca | 45.84 | 39.98 | 3.17 | 5.13 | 23.93 | 25.22 | 6.31 |
| Pseudochazara hippolyte | 41.73 | 47.85 | 4.06 | 5.35 | 30.43 | 34.58 | 7.5 |
| Pseudochazara mamurra | 50.01 | 52.21 | 7.22 | 8.28 | 30.86 | 36.05 | 5.74 |
| Pseudochazara mniszechii | 31.68 | 36.33 | 12.59 | 12.99 | 24.96 | 27.13 | 5.15 |
| Pseudochazara orestes | 33.12 | 39.72 | 11.48 | 10.35 | 26.12 | 29.02 | 7.29 |
| Pseudophilotes abencerragus | 40.58 | 39.92 | 6.76 | 2.89 | -12.44 | 2.78 | 15.72 |
| Pseudophilotes barbagiae | 43.26 | 38.61 | -5.99 | -7.13 | 18.14 | 24.54 | 7.99 |
| Pseudophilotes baton | 56.82 | 37.35 | 3.7 | 5.42 | -8.19 | -6.69 | 19.61 |
| Pseudophilotes bavius macedonicus | 30.68 | 30.84 | -3.5 | -3.45 | 6.57 | 6.48 | 0.19 |
| Pseudophilotes panoptes | 51.75 | 45.03 | 5.1 | 4.71 | -12.78 | -9.35 | 7.56 |
| Pseudophilotes vicrama | 54.49 | 39.1 | 3.6 | 0.94 | -12.92 | 1.23 | 21.08 |
| Pseudotergumia fidia | 21.18 | 30.91 | 2.21 | -1.27 | 13.82 | 17.06 | 10.83 |
| Pseudotergumia wyssii wyssii | 33.01 | 34.48 | 1.83 | -0.49 | 24.9 | 22.34 | 3.75 |
| Pyronia bathseba | 31.21 | 43.46 | 19.02 | 21.18 | 21.7 | 32.38 | 16.39 |
| Pyronia cecilia | 40.4 | 45.67 | 16.28 | 17.64 | 25.99 | 30.71 | 7.2 |
| Pyronia janiroides | 36.08 | 47.62 | 10.81 | 8.98 | 32.74 | 37.05 | 12.45 |
| Pyronia tithonus | 29.48 | 39.15 | 14.54 | 16.48 | 18.84 | 27.77 | 13.3 |
| Quercusia quercus | 24.6 | 19.36 | 9.47 | 6.21 | -14.96 | -1.12 | 15.15 |
| Satyrium acaciae | 33.66 | 25.94 | 1.88 | 3.3 | 11.78 | 8.71 | 8.44 |
| Satyrium esculi | 21.38 | 28.49 | 3.34 | 0.1 | 8.89 | 10.32 | 7.95 |
| Satyrium ilicis | 21.51 | 27.08 | 3.37 | 3.34 | 6.47 | 10.52 | 6.89 |
| Satyrium ledereri | 33.21 | 32.02 | 2.76 | 3.68 | 11.58 | 13.97 | 2.82 |
| Satyrium pruni | 23.12 | 26.37 | 8.74 | 10.45 | 15.84 | 18.19 | 4.36 |
| Satyrium spini | 29.1 | 30.55 | 3.94 | 5.33 | 13.99 | 14.26 | 2.03 |
| Satyrium w-album | 19.73 | 26.44 | 6.46 | 8.95 | 11.64 | 16.74 | 8.79 |
| Satyrus actaea | 14.01 | 31.63 | 3.94 | 8.22 | 9.9 | 25.31 | 23.8 |
| Satyrus ferula | 15.18 | 28.86 | 4.87 | 10.43 | 11.51 | 23.98 | 19.33 |
| Scolitantides orion lariana | 26.83 | 29.18 | -5.93 | -5.95 | 5.63 | 16.74 | 11.36 |
| Tarucus balcanicus | 46.56 | 52.28 | 8.5 | -0.61 | 10.89 | 34.4 | 25.85 |
| Tarucus rosaceus | 53.46 | 55.5 | 9.93 | 1.61 | 9.21 | 30.15 | 22.63 |
| Tarucus theophrastus | 49.98 | 41.4 | 9.58 | 0.53 | 2.57 | 21.31 | 22.51 |
| Thecla betulae | 21.57 | 27.16 | 3.47 | 8.89 | 8.91 | 14.27 | 9.45 |
| Tomares ballus | 42.64 | 48.52 | 4.92 | 14.07 | 20.2 | 34.73 | 18.15 |
| Tomares mauretanicus | 32.11 | 47.4 | 9.39 | 11.96 | 19.19 | 33.74 | 21.26 |
| Tomares nogelii | 31.87 | 47.57 | 14.38 | 15.92 | 21.45 | 37.6 | 22.57 |
| Turanana endymion | 33.02 | 32.99 | -1.39 | -1.43 | 16.82 | 16.81 | 0.06 |
| Turanana endymion taygetica | 39.01 | 38.87 | 10.82 | 10.97 | -22.06 | -22.36 | 0.36 |
| Ultraaricia anteros | 56.45 | 33.61 | -4.45 | 10.06 | -4.68 | 21.97 | 37.98 |
| Vacciniia opilete | 30.96 | 38.39 | 19.79 | -2.97 | -38.15 | 8.99 | 52.87 |
| Vanessa atalanta | 20.55 | 20.52 | 7.56 | 7.55 | 12.92 | 12.89 | 0.05 |
| Vanessa cardui | 35.27 | 35.39 | 11.19 | 11.16 | 28.41 | 28.36 | 0.13 |
| Vanessa indica | 25.62 | 25.55 | 9.88 | 9.74 | 20.45 | 20.28 | 0.23 |
| Vanessa virginiensis | 33.5 | 33.61 | 13.75 | 13.8 | 28.94 | 29.1 | 0.2 |
| Ypthima asterope | 28.35 | 31.48 | 3.36 | 3.46 | 16.01 | 15.94 | 3.14 |
| Zegris eupheme | 81.92 | 84.32 | -4.33 | -2.42 | 21.22 | 15.27 | 6.69 |
| Zerynthia cerisy | 63.69 | 48.72 | -5.85 | -4.66 | 41.83 | 25.38 | 22.28 |
| Zerynthia cerisy cerisy | 45.61 | 45.56 | -2.78 | -2.76 | 28.79 | 28.75 | 0.07 |
| Zerynthia polyxena | 48.05 | 47.23 | -1.73 | -1.86 | 36.25 | 35.86 | 0.92 |
| Zerynthia polyxena ochracea | 37.18 | 36.97 | 8.54 | 8.5 | 33.29 | 33.17 | 0.25 |
| Zerynthia rumina | 39.93 | 40.43 | -0.73 | -2.46 | 30.25 | 26.89 | 3.81 |
| Zerynthia rumina canteneri | 35.43 | 35.18 | 5.85 | 5.82 | 31.72 | 31.53 | 0.32 |
| Zerynthia rumina honnoratii | 46.24 | 46.14 | 2.34 | 2.32 | 30.99 | 30.93 | 0.12 |
| Zerynthia rumina medesicaste | 42.14 | 41.64 | -0.74 | -0.75 | 29.3 | 28.89 | 0.65 |
| Zizeeria knysna | 32.68 | 31.21 | 10.84 | 0.85 | -18.76 | 1.75 | 22.87 |

## Figures

Figure S1: Comparison between the structure in color space using either photographs or drawings for a subset of 53 species. Each point is a represents a color distance between two specimens (across species and sexes). See methods for more details. The three plots refer to the three different color axes (L, a and b).

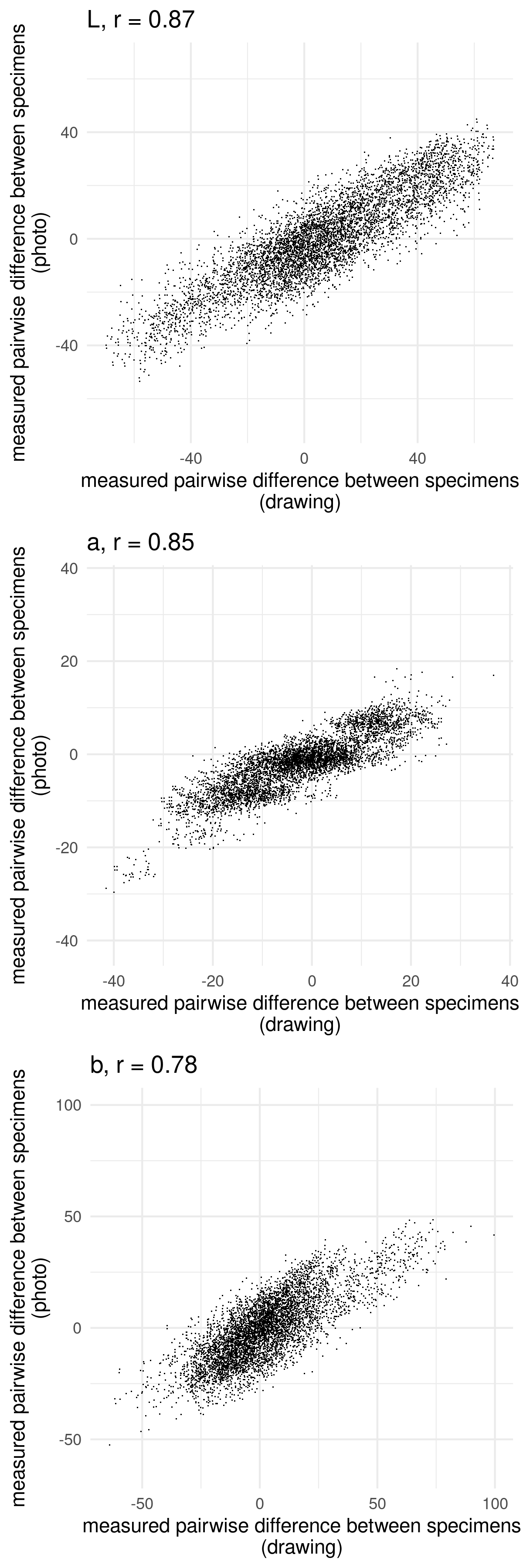


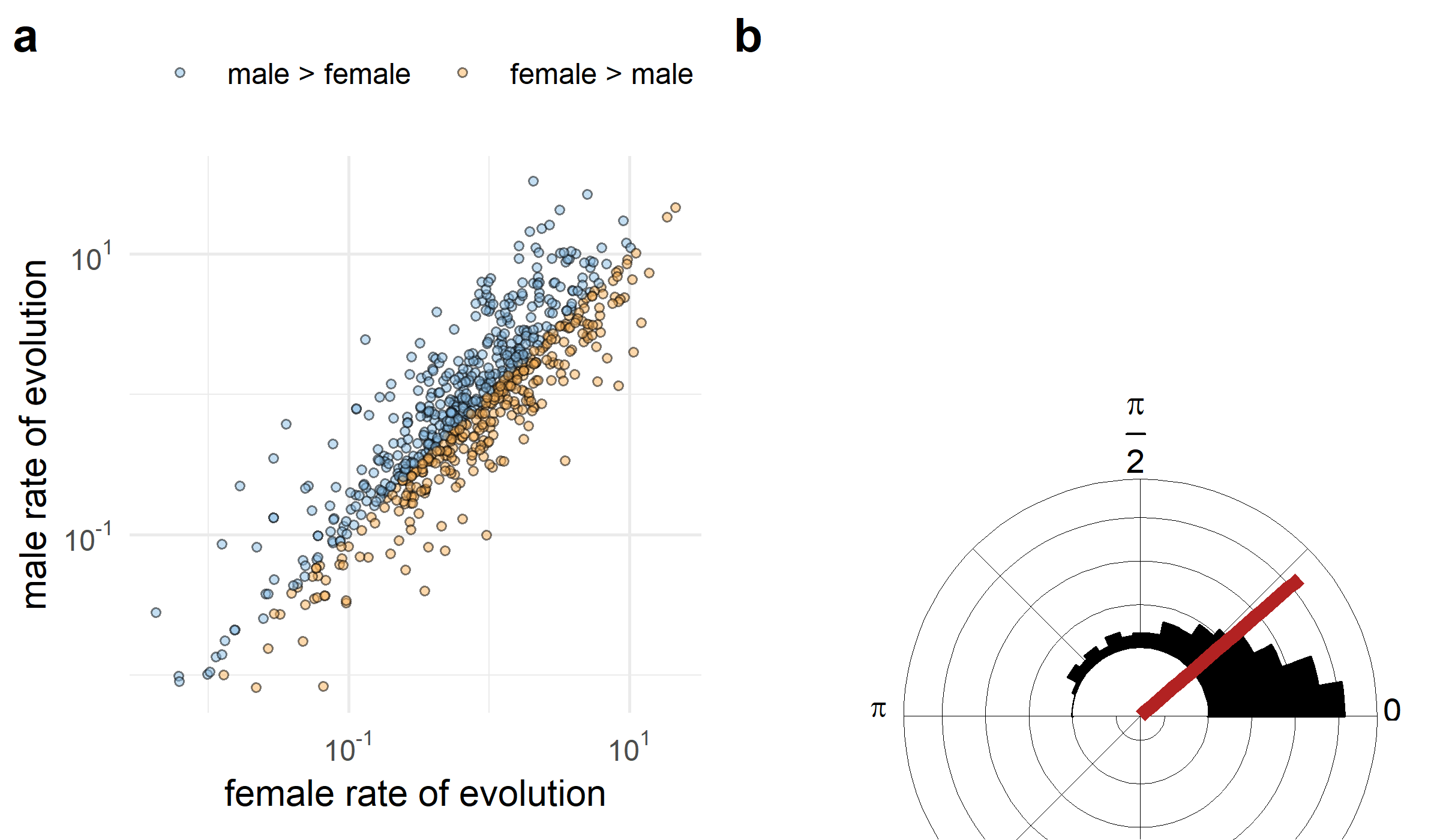


Figure S2: Male and female rates of butterfly color evolution (dorsal side) a While male color tends to evolve faster, evolutionary rates are generally highly correlated. b Male and female color is generally closely aligned and evolve in the same direction (angle 0 represents perfect alignment, angle π represent directly opposed). The red line indicates the average angle.


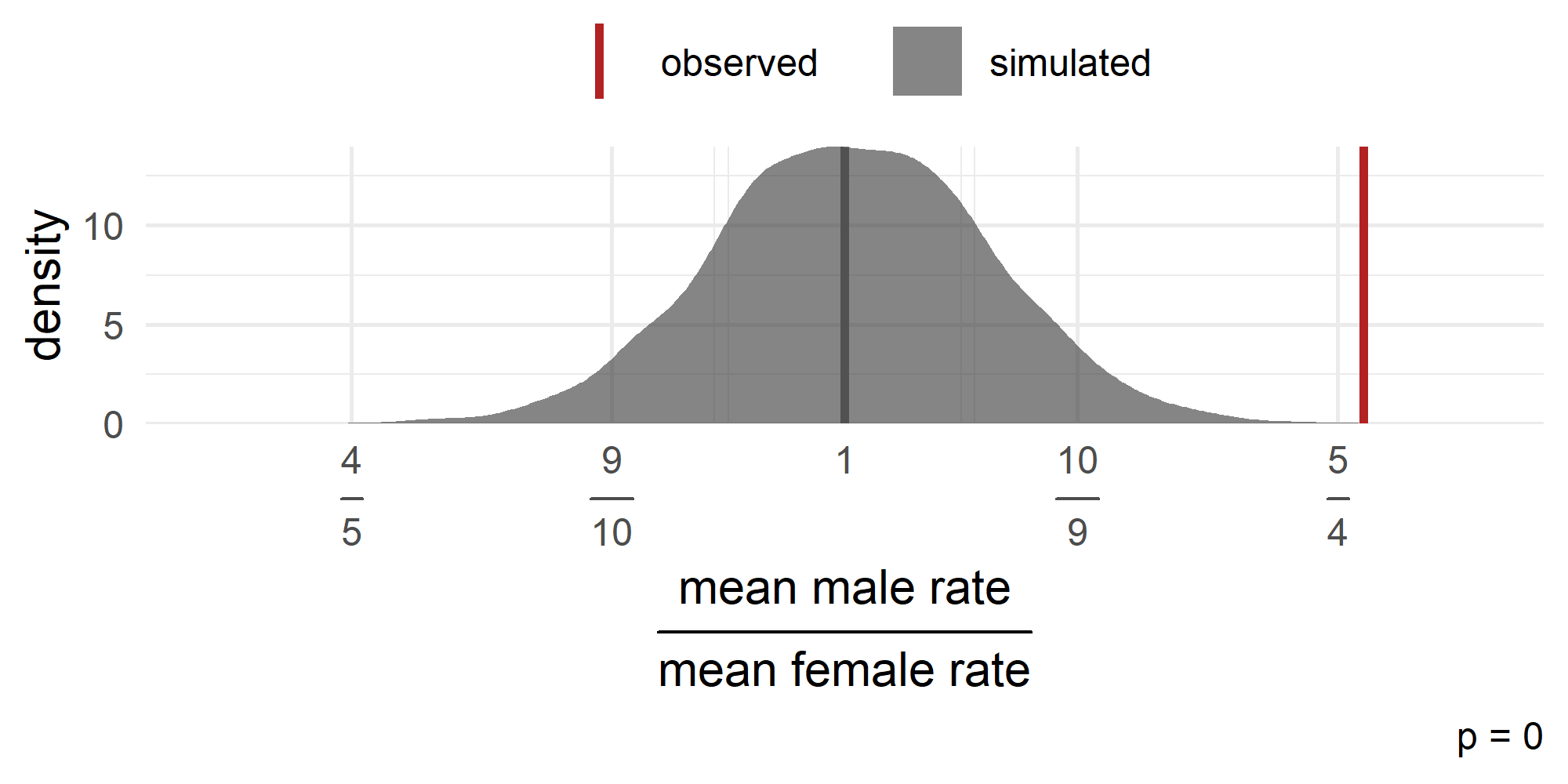


Figure S3: Comparison of the observed ratio between male and female evolutionary rate with the expected distribution from permuted phenotypes (see main text). P < 0.001.


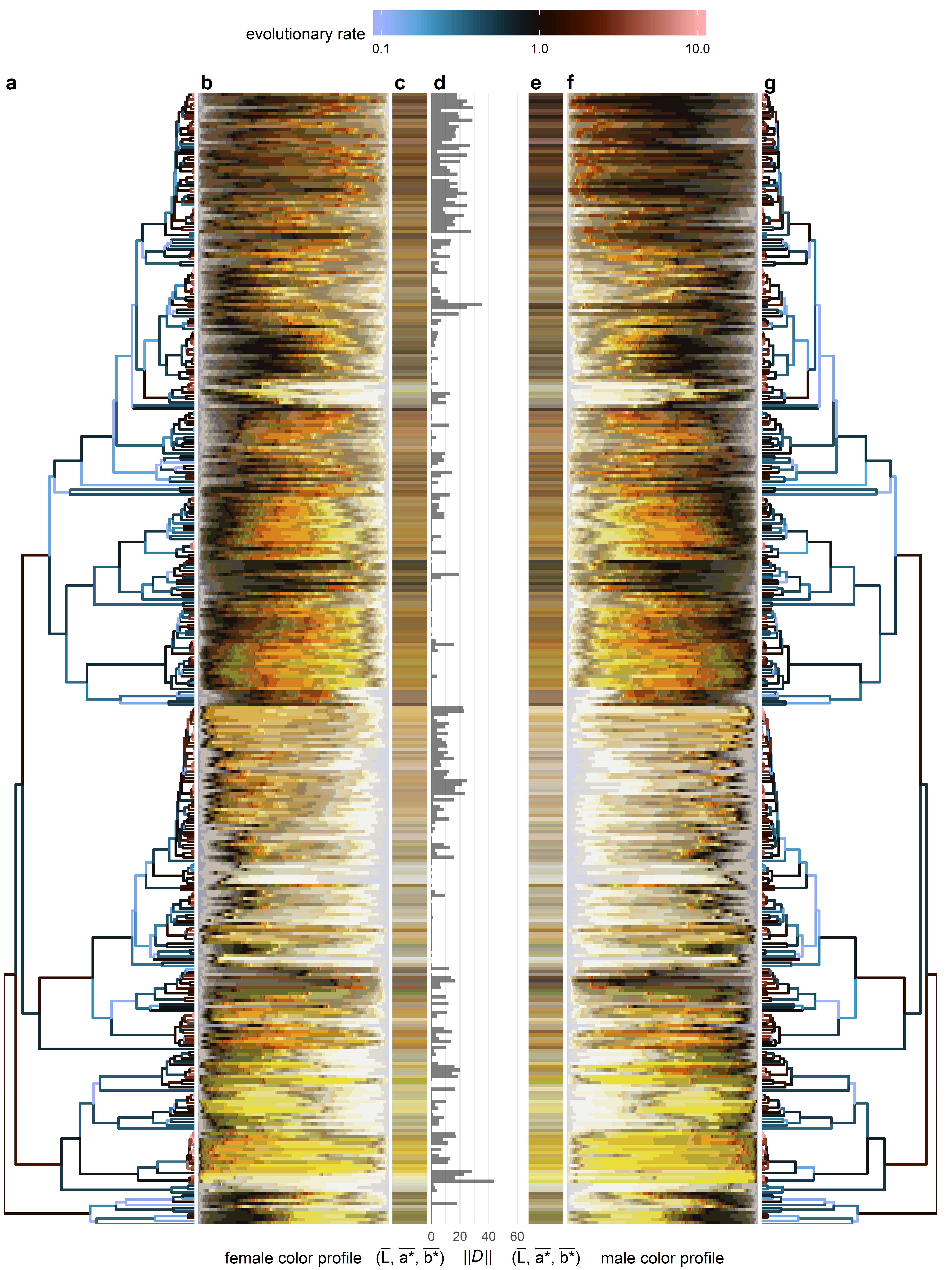


Figure S4: Coloration by sex of European butterflies, on the ventral sides. Panels a and g depict the phylogenetic relationships between species, with the branches colored by the estimated evolutionary rate for females and males respectively. Panels b and f illustrate the color profiles of each sex, respectively, for each species as the fraction of pixels in each of 50 color clusters. c and e give the female and male color centroids (average color) in Lab color space. Panel d plots the distance between the female and male color centroids, used as the metric for dichromatism.


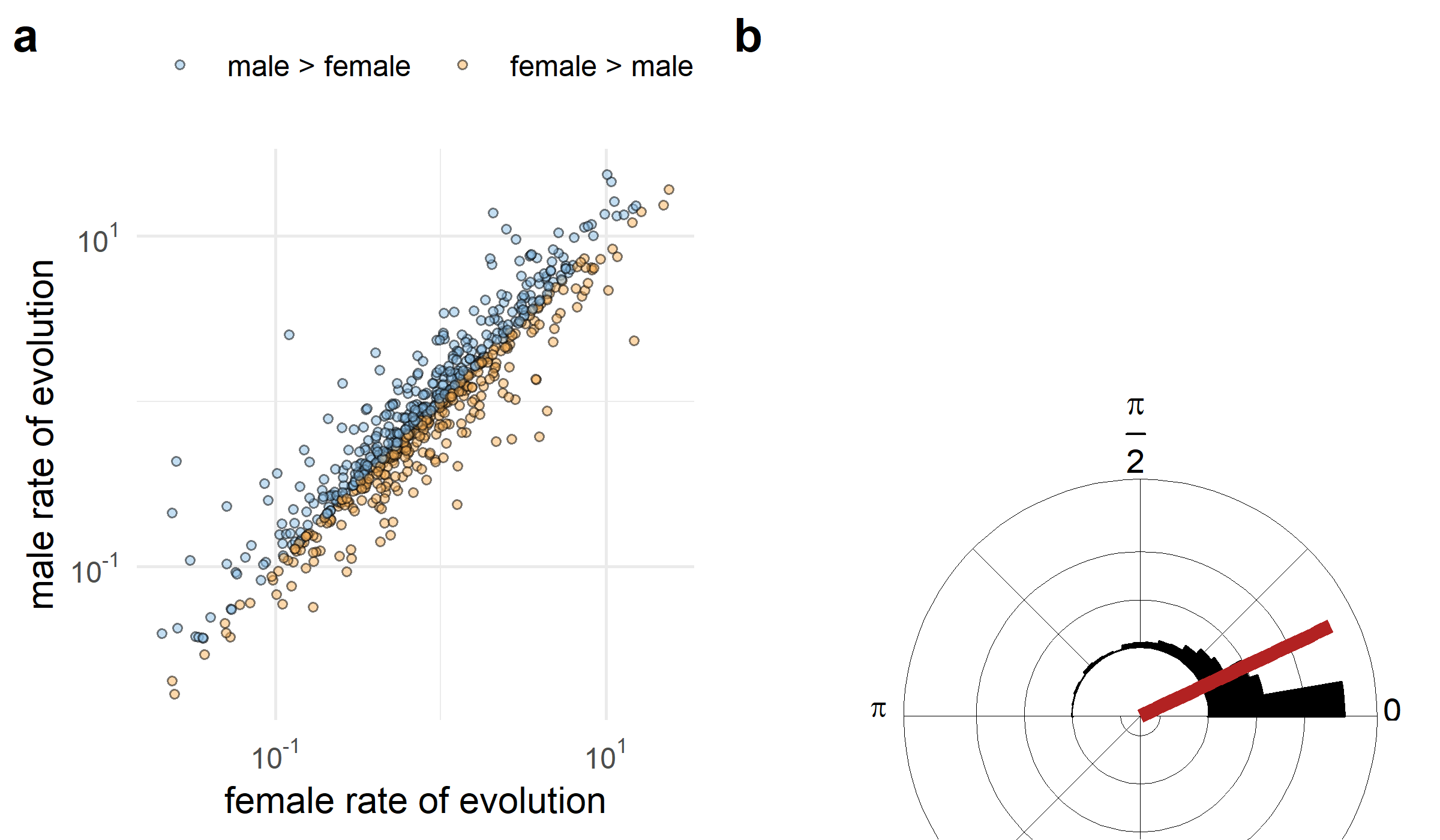


Figure S5. Male and female color evolution are strongly correlated, also on the ventral sides. a While male color tends to evolve slightly faster (see figure S5), evolutionary rates are generally highly correlated. b Male and female color is generally closely aligned and evolve in the same direction (angle 0 represents perfect alignment, angle π represent directly opposed). The red line indicates the average angle.


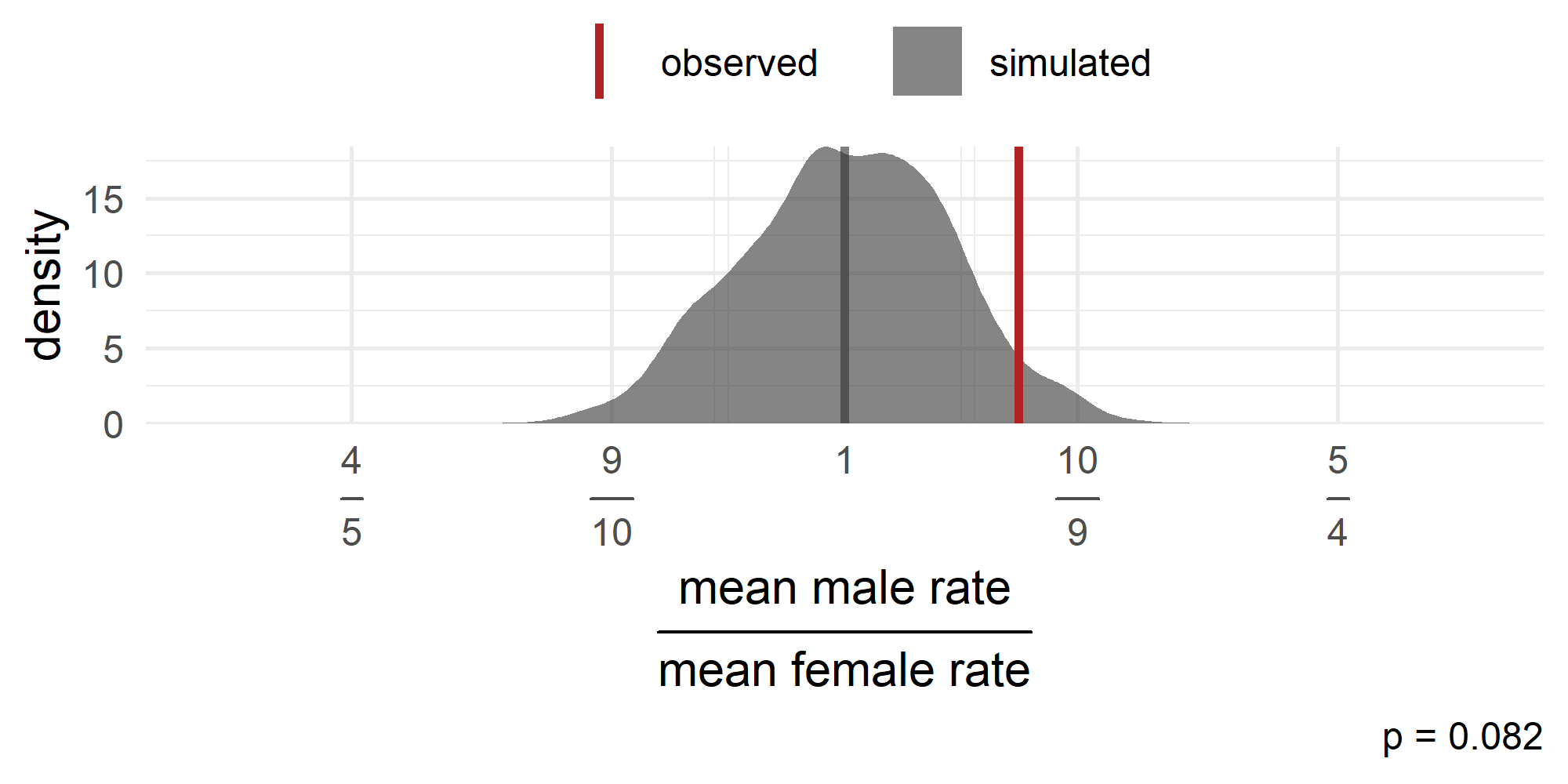


Figure S6: Comparison of the observed ratio between male and female evolutionary rate with the expected distribution from permuted phenotypes (see main text) for the ventral sides. P = 0.082.


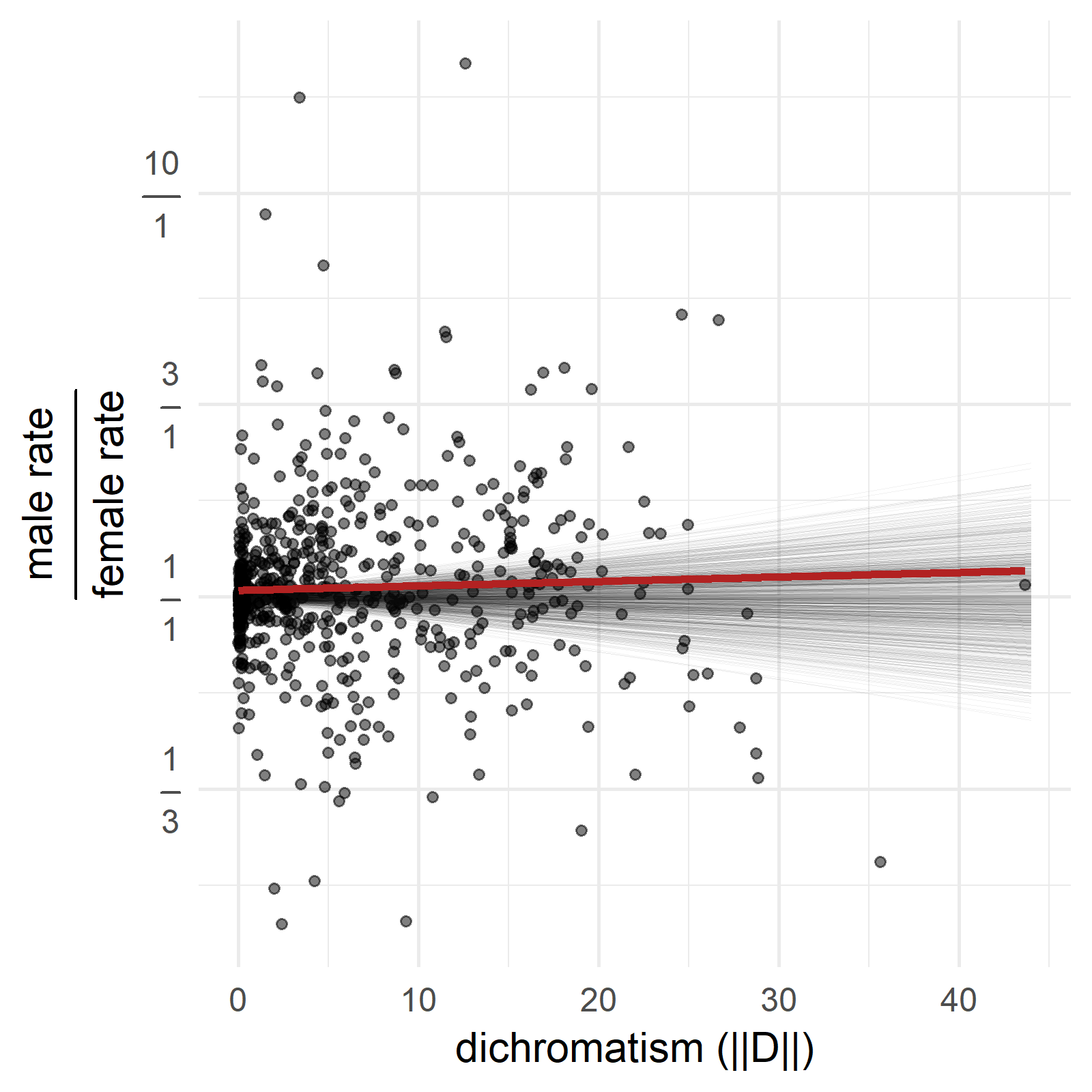


Figure S7: As ventral side dichromatism increases color evolution becomes evenly balanced between males and females. The points indicates the dichromatism and ratio of male to female evolutionary rate in color evolution of each branch in the phylogeny. The red line shows the observed relationship, while each thin grey line represents the slope of that relationship in each of 1000 permutations.


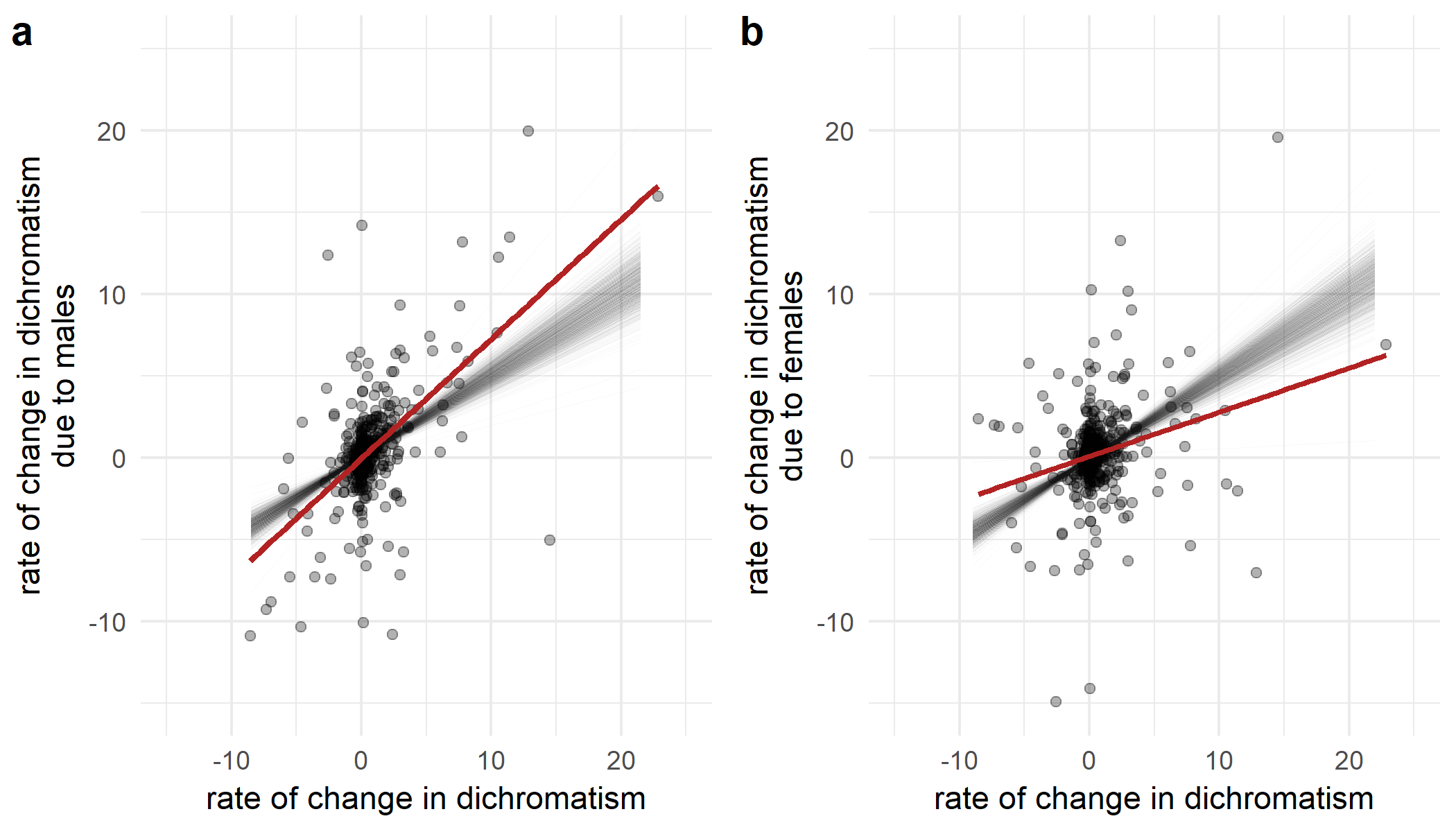


Figure S8: Changes in ventral side dichromatism are more likely to be the result of male change than female change. The x-axis represents effective rates of change in dichromatism, for each species. The y-axis shows the male (a) or female (b) attributable parts of that change along the same branch, which is the evolutionary rate of color evolution in the direction of dichromatism (see main text). The red lines shows the observed relationships, while each thin grey line represents the slope of that relationship in each of 1000 permutations. Note that the results in the two panels are not independent, the slopes necessarily add up to 1.


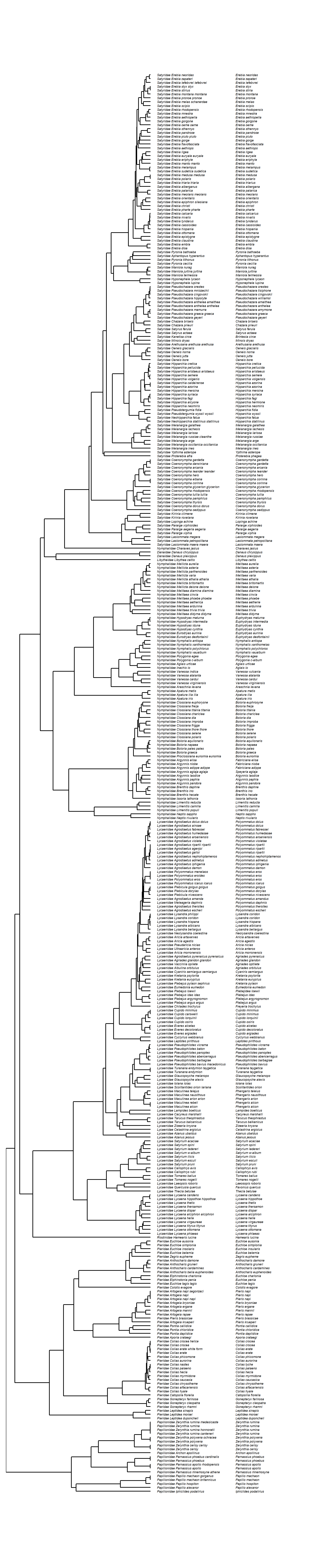


Figure S9: The phylogenetic tree as used in this study, identical to figure 2. Added as tip labels are the species names. On the left are the names as used in the source material (Tolman & Lewington, 1997). On the right are current binomial names.
